# All-optical closed-loop control of human cardiomyocyte networks exploiting holographic optogenetics

**DOI:** 10.1038/s44172-026-00779-1

**Published:** 2026-09-17

**Authors:** Robert Wendland, Felix Schmieder, Muhammad Atif Sikandar, Frithjof Paul Knüppel, Wolfram-Hubertus Zimmermann, Olaf Bergmann, Lars Büttner, Jürgen W. Czarske

**Affiliations:** 1 Laboratory of Measurement and Sensor System Techniques, TUD Dresden University of Technology, Dresden, Germany; 2 Competence Center BIOLAS, TUD Dresden University of Technology, Dresden, Germany; 3 Department of Pharmacology and Toxicology, University Medical Center Göttingen, University Medical Center Göttingen, Göttingen, Germany; 4 TUD Dresden University of Technology, Dresden, Germany; 5 Deutsches Zentrum für Herz-Kreislauf-Forschung (DHKZ) partner site Lower Saxony, Göttingen, Germany; 6 Cluster of Excellence “Multiscale Bioimaging: from Molecular Machines to Networks of Excitable Cells” (MBExC), University of Göttingen, Göttingen, Germany; 7 German Center for Neurodegenerative Diseases (DZNE), Göttingen, Germany; 8 German Center for Child and Adolescent Health (DZKJ), Göttingen, Germany; 9 Fraunhofer Institute for Translational Medicine and Pharmacology (ITMP), Göttingen, Germany; 10 Karolinska Institutet, CMB, Stockholm, Sweden; 11 Wyant College of Optical Sciences, University of Arizona, Tucson, AZ USA; 12 Institute of Applied Physics, Faculty of Science, TUD Dresden University of Technology, Dresden, Germany; 13 Excellence Cluster Physics of Life, TUD Dresden University of Technology, Dresden, Germany

**Keywords:** Optical imaging, Tissue culture, Image processing

## Abstract

Heart diseases can trigger life-threatening arrhythmias by disrupting cardiac contraction wavefront propagation. In contrast to conventional treatments like electrical pacemakers, optogenetics offers high spatial and temporal resolution for non-invasive interrogation and modulation of cellular activity. However, combining sensing and actuation into a real-time control loop remains challenging. A major obstacle is the data load associated with dense sensor arrays typically required for spatially resolved wavefront monitoring. To overcome this, we present an all-optical, closed-loop control strategy using adaptive holographic stimulation and sparsely sampled, label-free imaging data, enabling robust classification and quantitative characterization of spatial wavefront properties in real time. We successfully demonstrate spatiotemporal optogenetic closed-loop control of wavefront dynamics in functional syncytia of human induced pluripotent stem cell-derived cardiomyocytes in at least 107 out of 109 evaluated cases across all experiments. This framework establishes a foundation for advanced feedback control strategies aimed at mimicking, detecting and terminating abnormal propagation patterns.

## Abstract (web summary)

Robert Wendland and colleagues present an all-optical platform that combines sparse, label-free sensing of contraction wavefronts in cardiomyocyte syncytia with adaptive holographic optogenetic stimulation. The system enables real-time classification and spatiotemporal closed-loop control of wavefront propagation, e.g., for the testing of cardioversion strategies.

## Introduction

Heart diseases remain the leading cause of death worldwide^1^. A central aspect of cardiac dysfunction is the disruption of the normal cardiac rhythm, which can compromise oxygen supply and affect the functionality of the cardiovascular system^2–4^. Such disturbances, including tachycardia, are associated with characteristic spatiotemporal patterns of electrical activation in cardiac tissue. These alterations in electrical activation can arise from different underlying mechanisms. In focal tachycardia or focal-like activation, excitation originates from a localized source outside the physiological pacemaker region, accelerating the heart rate^5^. In contrast, reentrant tachycardia can be a life-threatening condition with heart rates typically above 100 bpm^2,6,7^, sustained by rotating electrical excitation wavefronts. Such reentrant activity can be observed as spiral wave patterns in simplified tissue preparations^8–14^. These arise from conduction velocity heterogeneities and the presence of electrically unexcitable regions such as scar tissue, both of which are promoted by adverse remodeling^3,4,15,16^. At the whole-heart level, the relationship between atrial and ventricular activation is complex due to conduction through the atrioventricular node, potentially leading to partial or complete decoupling of atrial and ventricular activation. As a result, atrial tachyarrhythmias do not necessarily translate into altered ventricular activation patterns, which primarily determine cardiac contraction. While focal and reentrant tachycardias are clinically defined at the organ level, these dynamics can be represented by focal-like activation and reentrant wave propagation in simplified in vitro systems such as cardiomyocyte monolayers. The present study focuses on the underlying local activation dynamics that give rise to these patterns, as understanding these dynamics is crucial for the development of targeted therapeutic strategies. In this context, optogenetics has emerged as a powerful tool for arrhythmia research^17–21^. By introducing light-sensitive ion channels into cardiomyocytes, optogenetics enables activation or inhibition of cellular activity with high spatiotemporal resolution^22^. Optical approaches offer significant advantages over conventional electrical stimulation, including the ability to target multiple regions simultaneously in three dimensions, to modulate stimulation or inhibition intensity, and to reduce pain and tissue damage^22–26^. These unique properties have motivated research in optical pacemaking and cardioversion approaches, where patterned light is utilized to terminate arrhythmic activity and restore normal rhythm^17–21,27,28^. Although such studies have demonstrated the feasibility of optogenetic cardioversion approaches, they were performed in open-loop configurations. Closed-loop systems would enable feedback-controlled studies of wavefront interactions, possibly improving our understanding of arrhythmia mechanisms and supporting the investigation of optical cardioversion strategies. Optogenetic closed-loop approaches were initially developed for the control of neural activity^29^ and have since been successfully transferred to cardiac cells^30^ and tissue-level preparations^18,31,32^. In this context, Scardigli et al. demonstrated an optogenetic closed-loop approach based on feedback from two temporal signals^18,33^. While this demonstrated real-time intervention in principle, it did not address the ongoing spatial characteristics of the underlying activation patterns. Because the efficacy of targeted cardioversion depends on correctly identifying the underlying mechanism^5,21,34^, systems must discriminate between them and adapt the stimulation pattern accordingly.

To bridge this gap, we present an all-optical platform for label-free monitoring and optogenetic stimulation with high spatiotemporal precision. We use the mechanical deformation of the cardiomyocytes during contraction as a surrogate for electrical activation, since cardiac excitation is physiologically coupled to mechanical contraction via excitation-contraction coupling^35^. Using our system, we establish a minimally invasive closed-loop cardioversion approach that relies on sparse spatial sampling of time traces derived from local cardiomyocyte contraction, while enabling control of the spatial properties of propagating wavefronts. We demonstrate our system by optogenetically inverting registered wavefronts in human induced pluripotent stem cell-derived cardiomyocytes (hiPSC-CMs) expressing the light-gated ion channel f-Chrimson^36,37^. Using hiPSC-CMs to complement animal models, our system therefore holds promise for both optogenetics-enhanced modeling of cardiac diseases and the assessment of possible optogenetic cardioversion strategies.

## Results

### Optical Setup

**Fig. 1:**
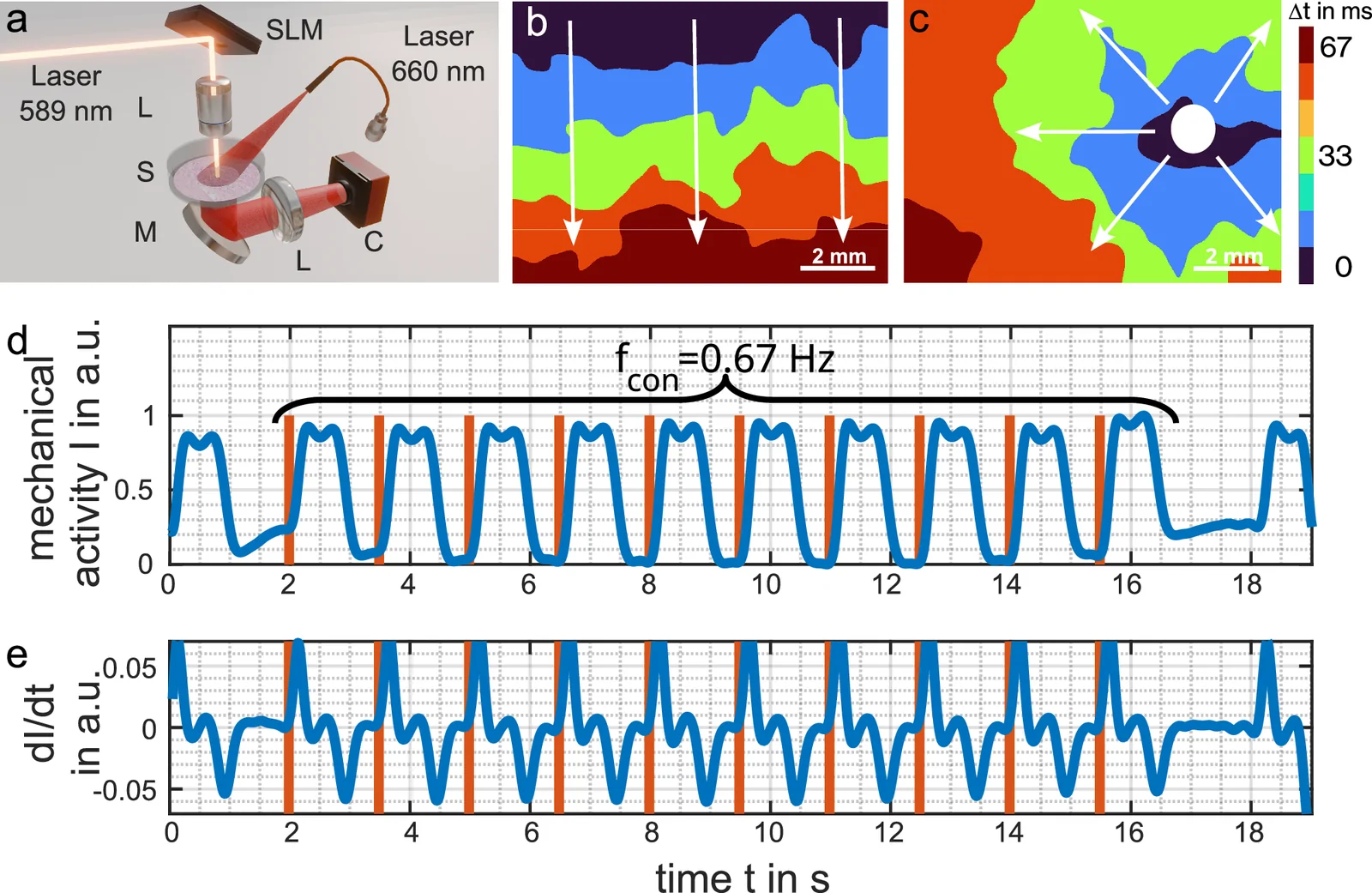
All-optical label-free stimulation and open-loop control. **a** Simplified scheme of the optical setup for stimulation (orange path) and imaging (red path) of the hiPSC-CMs. Components: SLM spatial light modulator, L lens, S sample, M mirror, C camera. Spatiotemporal stimulation patterns are realized by a holographic stimulation approach based on Fourier holograms. For label-free imaging, an obliquely mounted laser beam is used, resulting in speckle patterns on the camera. The schematic was created using assets from the Optical Components Pack v1 by Ryo Mizuta Graphics. **b** Isochrones of spontaneous planar contraction without optical stimulation. **c** Isochrones of a spherical contraction induced by optical stimulation; the white dot indicates the stimulation area (${P}_{{{\rm{stim}}}}=0.98$ mW; ${t}_{{{\rm{stim}}}}=90$ ms; ${A}_{{{\rm{stim}}}}=1.2$ mm^2^). Arrows indicate wavefront propagation direction. **d** Normalized mechanical activity I obtained from speckle patterns; orange lines highlight the stimulation time stamps. ${f}_{{{\rm{con}}}}$ denotes the measured contraction frequency. **e** Temporal derivative ${dI}/{dt}$ of (**d**). Maxima of the derivative coincide with the optical stimulation.

To enable non-invasive investigations of cellular behavior, we developed an all-optical, label-free setup comprising of a stimulation and an imaging part, as illustrated in Fig. 1a. For stimulation, a 589 nm laser was chosen to match the action spectrum peak of the opsin f-Chrimson^36–38^. The laser beam is shaped by a ferroelectric SLM displaying computer-generated holograms (CGHs). The spatially and temporally modulated laser beam is Fourier transformed and projected onto the sample plane by a lens system, yielding stimulation patterns that allow for targeted illumination with cellular resolution in a field of view of approx. (19 × 9.5) mm^2^ at a maximum repetition rate of 1.7 kHz. This is necessary for the implementation of closed-loop control schemes in the range of several hundred Hertz on a significant part of the sample. To measure the mechanical response of the sample, we use a label-free imaging approach based on the method described by Burton et al. ^19^. The sample is illuminated with a 660 nm laser beam at an oblique angle, producing a speckle pattern that changes locally upon cell contraction and is recorded by a camera through a custom-built microscope. An example of such a speckle image and the corresponding widefield image is shown in Supplementary Fig. S1c,d. Via excitation-contraction coupling, the recorded mechanical deformation-induced intensity changes act as a time-delayed surrogate for electrical activation^39^. The corresponding delay between the electrical and mechanical signal is on the order of a few hundred milliseconds^40^. More detailed descriptions of the optical setup and the CGH calculation approach are given in the Methods section and Supplementary Note 1. Within this framework, imaging was implemented in a transmission configuration. However, contraction activity could also be measured in reflection from backward-scattered light, at the expense of reduced photon yield at the camera. This may prove particularly useful for label-free studies of mechanical activity in three-dimensional structures.

To analyze the cardiomyocytes’ contraction behavior, we developed data processing approaches to extract both the contraction-derived time signal and the spatial shape of the propagating contraction wavefronts from the recorded speckle patterns (see Methods 4.7, 4.8 and Supplementary Note 3, 4). Whole-image time signals were extracted by tracking the sum of local intensity changes with respect to a slowly varying mean background, followed by low-pass-filtering and normalization within a moving time window. To validate our approach, we first established an open-loop configuration, in which predefined stimulation patterns were applied at a fixed frequency and the resulting responses were analyzed post hoc. This allowed us to determine whether cardiomyocytes reliably followed the imposed pacing and to assess how specific spatial stimulation patterns modulated the shape of the emerging wavefronts. Figure 1d shows a representative time signal reflecting the contraction behavior of the cardiomyocytes, where orange lines mark the stimulation time stamps. Since the shape of the contraction signal may vary from peak to peak, we extract contraction frequencies from the maxima of the derivative of the contraction signal as outlined in Fig. 1e. It can be seen that the cells in this case show successful 1:1-coupling between stimulation and contraction, evidenced by two observations: Each stimulation pulse of the sequence is followed by a contraction and the stimulation alters the contraction frequency, confirming that the observed frequency match is not coincidental but rather causally linked to the stimulation. A detailed investigation of the stimulation frequency response is presented in Supplementary Note 2. Beyond monitoring the global mechanical activity of the cell cultures, the label-free imaging approach enables the visualization of propagating contraction wavefronts. This capability allows us to assess whether a stimulation with specific spatial patterns can modify the shape of the resulting wavefronts. The activity maps, i.e., isochrones of contraction, shown in Fig. 1b,c illustrate this capability: the spontaneously occurring planar contraction waves in Fig. 1b are converted into spherical wavefronts shown in Fig. 1c by projecting a dot-shaped stimulus onto the sample. This is also indicated by arrows showing the propagation direction of the wavefront.

These results underscore the potential of our all-optical, label-free system to control cardiomyocyte dynamics in both space and time. This provides a foundation for investigations into the optogenetic response of f-Chrimson-modified human cardiomyocyte networks (see Supplementary Note 2), as well as the realization and non-invasive evaluation of all-optical closed-loop control experiments (see section “*Spatiotemporal Closed-Loop Control of Cardiac Contraction Wavefronts Using Sparsely Sampled Data”*).

### Wavefront characteristics from sparsely sampled mechanical activity

**Fig. 2:**
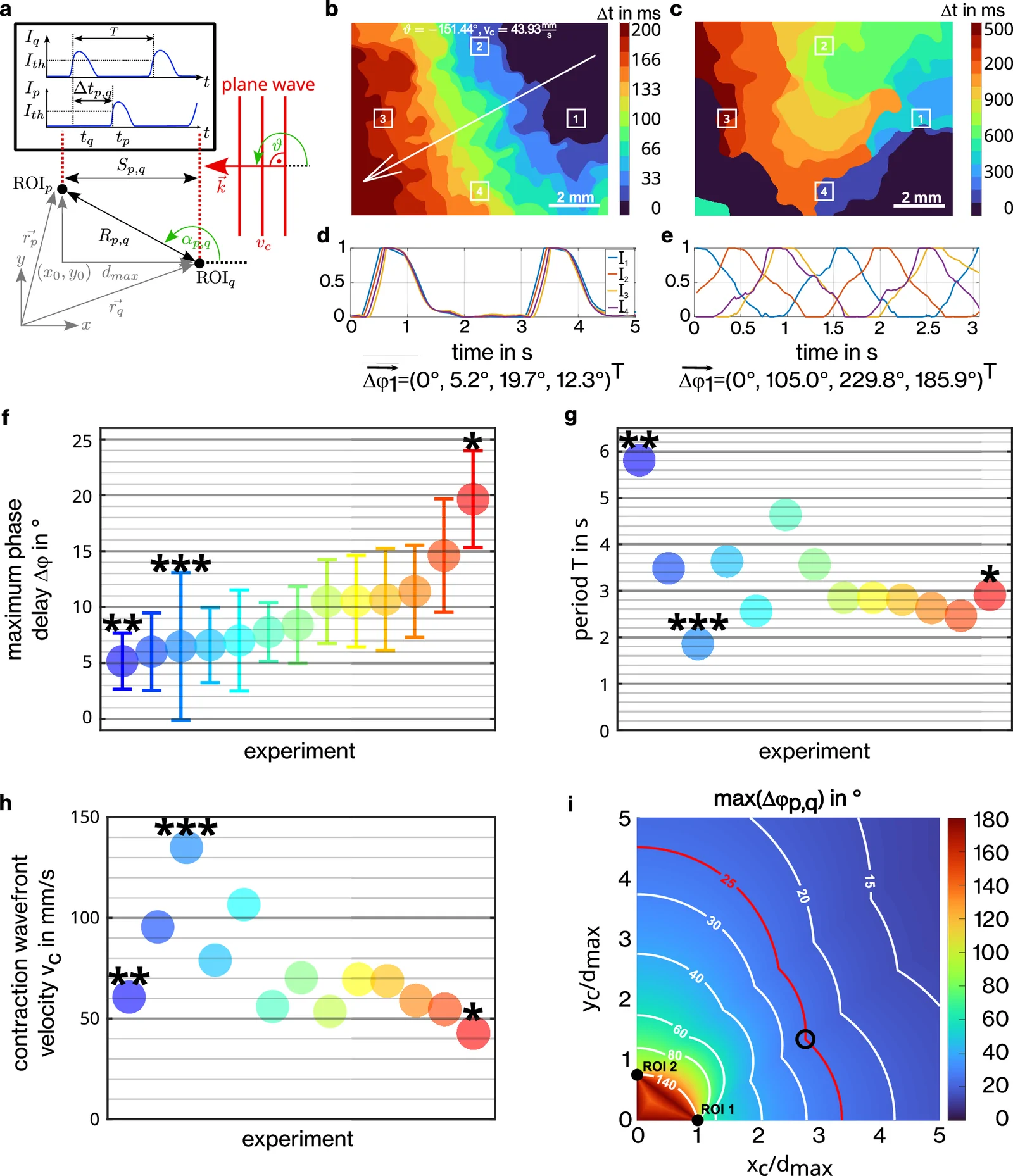
Determination of wavefront characteristics from regions of interest (ROIs). **a** Scheme of phase delay measurement between two ROIs p and q. ${I}_{p}$, ${I}_{q}$ denote the extracted contraction signal at ROIs over time t. ${t}_{p}$ and ${t}_{q}$ are the time stamps when the signal threshold ${I}_{{{\rm{th}}}}$ is passed at the respective region. $\triangle {t}_{p.q}$ denotes the temporal delay between these events. T is the contraction period. ϑ is the angle under which the plane wave (with wave vector $\vec{k}$) propagates with velocity ${v}_{{{\rm{c}}}}$. x and y are coordinates in the global coordinate system. ${\vec{r}}_{p}$ and ${\vec{r}}_{q}$ are vectors describing the position of ROI p and q within the global coordinate system. $\left({x}_{{{\rm{O}}}},{y}_{{{\rm{O}}}}\right)$ is the center of the ROI configuration. ${d}_{\max }$ denotes the maximum distance between $\left({x}_{{{\rm{O}}}},{y}_{{{\rm{O}}}}\right)$ and one ROI of the configuration. ${\alpha }_{p,q}$ denotes the angle of the direct connection between ROIs p and q. ${R}_{p,q}$ is the total length of this connection and ${s}_{p,q}$ the distance traveled by the wavefront in $\triangle {t}_{p.q}$. **b** Isochrone map of a plane contraction with four marked ROIs; the white arrow indicates the measured wave propagation direction. **c** Isochrone map of a spiral-shaped contraction with marked ROIs. **d**, **e** Signals corresponding to the contraction shown in (**b**, **c**), extracted from the ROIs. Spiral contractions show significantly larger phase delays ${\vec{\triangle \varphi }}_{1}$ (see Eq. 1) between ROI signals than plane waves. **f** Maximum phase delays between pairs of ROIs during spontaneous plane wave contractions; error bars represent the maximum possible deviation in phase delay due to inaccuracies in activation time detection; see Eq. 2 (each datapoint represents a single measurement) **g** Distribution of contraction periods. **h** Distribution of contraction wavefront velocities; identical datapoint colors across (**f**–**h**) indicate the same experiment. Symbols (*), (**) and (***) mark datapoints discussed in the text. **i** Maximum measured phase difference $\max \left(\triangle {\varphi }_{p,q}\right)$ over rotor core position $\left({x}_{{{\rm{c}}}},|,{y}_{{{\rm{c}}}}\right)$, normalized by the maximum distance ${d}_{\max }$ between an ROI and the ROI center point. ROIs are shown as black dots; isolines indicate the detection boundaries corresponding to different phase delay thresholds ${\triangle \varphi }_{{{\rm{th}}}}$; the red isoline marks the selected 25° threshold; the black unfilled circle marks the critical distance on the 25°-boundary with the shortest distance to the ROI center point.

The characterization of contraction wavefronts remains a challenge in optical implant research, since existing approaches typically rely on dense grids of signal sources^41–43^. The associated data volume renders spatiotemporal closed-loop control of contraction wavefronts practically infeasible^44^ and would require implants with thousands of electrodes covering the heart surface. To address this limitation, we propose an approach based on sparsely sampled signals. We realized the respective “sensors” optically by defining small regions of interest (ROIs) at selected positions. In each ROI, the contraction-derived time signal was acquired by extracting time signals from intensity fluctuations in the speckle patterns generated locally by the contracting samples. The temporal shifts between contraction activities at different sites provide direct information about wavefront characteristics. From this, the wavefront can be categorized as either rotating or planar, and in the latter case, its propagation direction and velocity can be determined. A scheme of the measurement geometry with relevant quantities is given for a planar contraction in Fig. 2a. The precision of the velocity and orientation measurement using sparsely sampled activity was evaluated with respect to different parameters like the number of ROIs in section “*Contraction Wavefront Velocity and Angle Measurement Using Sparsely Sampled Data”*. The algorithms used for signal extraction as well as orientation and velocity measurement are described in detail in Supplementary Note 5 and 6.

In Fig. 2b, the activation map of a planar wave propagation is shown, where the numbered white rectangles correspond to ROI 1 to 4. The respective time signals are shown in Fig. 2d. In this case, the signals are nearly in phase but for small time lags corresponding to the delayed activation connected with the contraction wavefront velocity and the propagation direction. The measured propagation direction is indicated by the white arrow as well as quantitatively given together with the measured velocity of the wavefront. When comparing the case of a plane wave propagation (Fig. 2b, d) with the case of a rotating spiral-shaped contraction (Fig. 2c, e), it is clearly visible that a distinction is possible based on the phase delays between the time signals. The phase shifts ${\vec{\triangle \varphi }}_{q}$ of each ROI signal ${I}_{p}$ relative to the arbitrarily chosen reference time signal of ROI 1 (q=1),

1 $${\vec{\Delta \varphi }}_{q}=\left(\begin{array}{c}\begin{array}{c}\Delta {\varphi }_{1,q}\\ \vdots \end{array}\\ \Delta {\varphi }_{p,q}\\ \vdots \end{array}\right),{{\rm{with}}}:\Delta {\varphi }_{p,q}=360^\circ \cdot \frac{\Delta {t}_{p,q}}{T}\,,$$

are given in Fig. 2d, e for the respective case. The temporal delay between the activity in ROIs p and q is denoted as $\Delta {t}_{p,q}$ and the phase $\Delta {\varphi }_{p,q}$ is calculated relative to the contraction period T. In the case of a rotating contraction pattern, much higher phase delays compared to the plane case occur (Fig. 2b, c). In the shown example, phase differences of up to 174.1° were observed. To determine a phase delay threshold for the wavefront characterization, we observed spontaneous plane contraction waves in N=13 independent cardiomyocyte networks and extracted time signals from ROIs, arranged as shown in Fig. 2b. Then, phase delays and the maximum phase delay between the four measured phases were calculated for every contraction according to Eq. 1. The results are plotted in Fig. 2f. In all of our experiments, the phase delay of a spontaneous plane wave remained below 19.7°. The measured phase delay is associated with an uncertainty. Its maximum relative error resulting from the finite sampling rate ${f}_{{{\rm{cam}}}}$ of the camera is given by (derivation provided in Supplementary Note 8):

2 $$\left|\frac{\Delta \left({\Delta \hat{\varphi }}_{p,q}\right)}{\Delta {\varphi }_{p,q}}\right|=\frac{2}{{f}_{{{\rm{cam}}}}}\cdot \left|\frac{{v}_{c}}{{R}_{p,q}\cdot \cos \left(\vartheta -{\alpha }_{p,q}\right)}+\frac{1}{T}\right| \, .$$

Here, ${R}_{p,q}$ and ${\alpha }_{p,q}$ are the distance and orientation angle between ROIs p and q and ϑ is the propagation angle of the plane wave as outlined in Fig. 2a. This error is represented by the error bars in Fig. 2f. Taking this error into account, we presume a phase delay threshold of ${\Delta \varphi }_{{{\rm{th}}}}=25^\circ$ for distinguishing a planar wave from a rotational one to be reasonable. This threshold strongly depends on the geometry of the ROI placement as well as on the contraction period (Fig. 2g) and the contraction wavefront velocity (Fig. 2h), as is qualitatively visible in Fig. 2f–h, where datapoints from the same experiment are shown in the same color. Particularly noteworthy are the datapoints with the maximum measured phase delay, highlighted with (*) across Fig. 2f–h and the one with the minimum phase delay (**). The large phase delay (*) results from a relatively low contraction wavefront velocity, while the low phase delay (**) is due to a long contraction period. Particularly emphasized is the measurement with the highest expected phase delay error, highlighted with (***) across Fig. 2f–h, resulting from a combination of short period and high contraction wavefront velocity. In this case, potential timing inaccuracies in detecting contractions in ROIs lead to increased deviations in the determined phase delay. Accordingly, we conclude that the measured phase delay of a planar wave increases with shorter periods T and lower contraction wavefront velocities ${v}_{c}$. Further, a short distance ${s}_{p,q}={R}_{p,q} \cdot \cos (\vartheta -{\alpha }_{p,q})$ (projection onto the propagation direction) between two ROIs p and q, leads to a lower measured phase delay. A summary of these dependencies and a comparison of measured phase delays across different experiments is provided in Supplementary Note 9.

We additionally investigated the boundary conditions under which a rotating contraction wave can still be distinguished from a plane wave. To assess the detection limit of our approach, a simple rotation model was used (see Supplementary Note 10), assuming an ROI alignment as illustrated in Fig. 2a: ROIs placed opposite each other around a center point, defined by the intersection of their connecting lines. In general, the maximum measured phase delay during a rotating contraction decreases when the rotation center moves outside the area between the ROIs. The critical rotor core distance ${d}_{{{\rm{crit}}}}$ from the ROI center marks the distance at which a rotating wave can no longer be distinguished from a planar wave. Denoting the longest distance from an ROI to the center ${d}_{\max }$ and assuming a detection threshold of ${\Delta \varphi }_{{{\rm{th}}}}=25^\circ$ as discussed above, the ratio ${d}_{{{\rm{crit}}}}/{d}_{\max }$ maximizes to approximately ${d}_{{{\rm{crit}}}}/{d}_{\max }=3.8$ for a square ROI alignment. Diverging from this arrangement results in smaller ${d}_{{{\rm{crit}}}}/{d}_{\max }$ as shown in Fig. 2i but may result in a higher ${d}_{{{\rm{crit}}}}$ when overcompensated by larger ROI distances. Furthermore, increasing the spacing between the ROIs reduces the error in phase delay measurement (see Eq. 2). The high ratio between the detectable rotor core distance and the maximum ROI distance ${d}_{{{\rm{crit}}}}/{d}_{\max }$ indicates the robustness of our ROI-based approach: even with a minimal number of sensors, it enables the detection of rotating waves far beyond the area directly covered by the ROI array or the camera’s field of view (Fig. 2i). Nevertheless, careful consideration must be given to the ROI array geometry and the selected phase delay threshold, as both parameters directly influence the spatial region in which rotational activity can be reliably distinguished from planar wave propagation. This poses a practical limitation when applying the method in experimental or clinical settings. The proposed real-time measurement approach using sparsely sampled signals of mechanical activity provides the foundation for optogenetic closed-loop control, as high temporal resolution is essential to respond to ongoing dynamics of contraction wavefronts with patterned light. Within our hardware framework, the ROI-based wavefront sensing works with up to 3.86 kHz. However, the camera employed for our experiments limits the achieved temporal resolution to 16.67 ms.

### Contraction wavefront velocity and angle measurement using sparsely sampled data

**Fig. 3:**
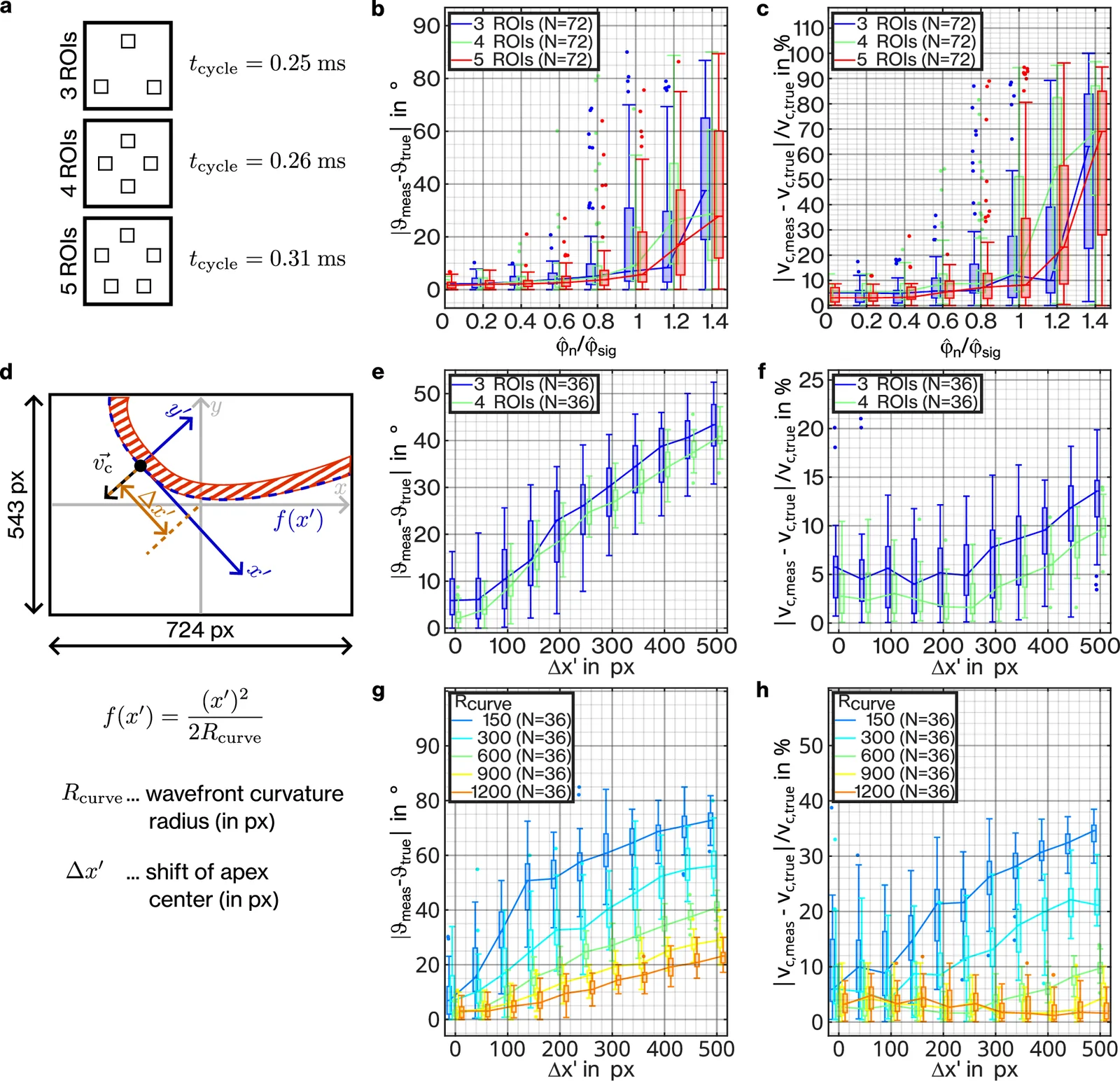
Simulative evaluation of robustness of the measurement approach against noise and deviations from ideal planar wave propagation. **a** Schematic overview of the evaluated ROI geometries and their corresponding achievable cycle times ${t}_{{{\rm{cycle}}}}$. **b** Uncertainty in wavefront orientation measurement as a function of different noise levels for different ROI configurations. The ratio ${\hat{\varphi }}_{{{\rm{n}}}}/{\hat{\varphi }}_{{{\rm{sig}}}}$ denotes the relative amplitudes of noise- and signal-induced phase changes in the sample plane. **c** Uncertainty in wavefront velocity measurement as a function of noise level for different ROI configurations. Measured values are denoted by ${\vartheta }_{{{\rm{meas}}}}$ and ${v}_{c,{{\rm{meas}}}}$, and prescribed simulation ground-truth values by ${\vartheta }_{{{\rm{true}}}}$ and ${v}_{c,{{\rm{true}}}}$. **d** Schematic of a non-ideal (curved) wavefront. x and y denote the coordinate axes of the field of view (FOV), and $\vec{{v}_{{{\rm{c}}}}}$ represents the propagation velocity vector. The apex of the curved wavefront is indicated by a black dot, with $x{\prime}$ and ${y}^{{\prime} }$ defining the corresponding local coordinate system. The displacement of the apex relative to the FOV center (also center of the ROI geometry) is denoted by $\triangle {x}^{{\prime} }.\,$The wavefront is modeled as a parabola with curvature radius ${R}_{{{\rm{curve}}}}$. **e** Uncertainty in wavefront orientation measurement as a function of apex displacement for a wavefront with curvature ${R}_{{{\rm{curve}}}}=600\,{{\rm{px}}}$, for different ROI configurations. **f** Uncertainty in wavefront velocity measurement under the same conditions as in (**e**). **g** Uncertainty in wavefront orientation measurement for varying apex displacements and wavefront curvatures. **h** Uncertainty in wavefront velocity measurement for varying apex displacements and wavefront curvatures. All simulated wavefronts had a propagation velocity of 80 mm/s. For all box plots: Boxes show the interquartile range (IQR) with the median indicated by the central line, whiskers extending to 1.5 × IQR, and individual points representing outliers; the solid lines connect the medians.

Besides the discrimination between planar and rotating contraction patterns, the ROI-based measurement approach enables the extraction of propagation direction and velocity of planar wavefronts. To assess the influence of different measurement parameters on the precision of velocity and angle for different ROI numbers, we established a simulation of the optical system based on Fourier optics (described in detail in Supplementary Note 7). First, the influence of noise, e.g., induced by external motion, was evaluated. This noise was modeled as a uniformly distributed random phase perturbation in the sample plane with a maximum amplitude of ${\hat{\varphi }}_{{{\rm{n}}}}$, relative to the maximum phase change associated with contraction-induced motion ${\hat{\varphi }}_{{{\rm{sig}}}}$. The precision of the ROI-based measurement depends on the propagation angle, due to the geometry of the ROI arrangement. Planar wave propagation was simulated for orientations ranging from 0° to 355° in steps of 5° with a velocity of 80 mm/s. A schematic overview of the evaluated ROI geometries and their corresponding cycle times for closed-loop operation is shown in Fig. 3a. Figure 3b presents the absolute propagation angle uncertainty for the different ROI geometries as a function of the ratio between maximum noise and signal amplitudes. For low noise levels (${\hat{\varphi }}_{{{\rm{n}}}}/{\hat{\varphi }}_{{{\rm{sig}}}}\le 0.6$), the mean angular inaccuracy remains below 5° for all investigated geometries. At higher noise levels (${\hat{\varphi }}_{{{\rm{n}}}}/{\hat{\varphi }}_{{{\rm{sig}}}} > 0.6$), the uncertainty increases more rapidly for all configurations. A slight performance advantage of the 5-ROI arrangement can be observed, although the mean absolute uncertainty exceeds 20° at higher noise levels. A similar trend is observed for the absolute velocity uncertainty (Fig. 3c). While the inaccuracy again increases more strongly for ${\hat{\varphi }}_{{{\rm{n}}}}/{\hat{\varphi }}_{{{\rm{sig}}}} > 0.6$, no clear advantage of the 5-ROI configuration is apparent.

It should be noted that these results represent a simulation-based assessment of methodological limitations. A ratio of ${\hat{\varphi }}_{{{\rm{n}}}}/{\hat{\varphi }}_{{{\rm{sig}}}}=1$ corresponds to a signal-to-noise ratio of 0 dB. This represents a substantially lower signal-to-noise ratio, i.e. higher relative noise, than in our experiments, where ${\hat{\varphi }}_{{{\rm{n}}}}/{\hat{\varphi }}_{{{\rm{sig}}}}=0.23\pm 0.11$ (mean ± standard deviation, N=12, see Supplementary Note 7). Overall, increasing the number of ROIs from three to five does not significantly improve robustness against noise. Nevertheless, under practically relevant conditions $({\hat{\varphi }}_{{{\rm{n}}}}/{\hat{\varphi }}_{{{\rm{sig}}}}\le 0.5)$, the angle and velocity deviations for all investigated ROI configurations remained well below 10° and 10 %, respectively, demonstrating robust performance when externally induced motion can be minimized, for example by using a damped optical table.

Since the measurement approach is based on the assumption of planar wave propagation, it is important to assess its sensitivity to deviations from this ideal case. To this end, two parameters were varied in the simulation: the curvature of the wavefront and the displacement of the curvature center (apex) relative to the center of the field of view (FOV), as illustrated in Fig. 3d. For orientation, the shown wavefront has a curvature of ${R}_{{{\rm{curve}}}}\approx 190\,{{\rm{px}}}$ and an apex shift of $\Delta x{\prime} \approx 170\,{{\rm{px}}}$. A comparison of the 3- and 4-ROI configurations for a wavefront with a curvature radius of 600 px and varying apex shifts is visualized in Fig. 3e, f. For most evaluated cases, the 4-ROI configuration exhibited a mean uncertainty in wavefront orientation that was approximately 3° lower than that of the 3-ROI arrangement. In addition, the standard deviation was substantially reduced, yielding a more reliable estimation of the propagation angle. Similarly, the 4-ROI configuration consistently yielded lower errors in propagation velocity across all investigated apex shifts. Overall, both configurations show comparable trends, with increasing uncertainties for larger apex shifts. While the uncertainty in orientation determination increases approximately linearly with apex displacement, the error in velocity estimation remains nearly constant up to an apex shift of 250 px. Based on its better performance, the 4-ROI configuration was selected for further analysis as a compromise between accuracy, robustness to non-planar wavefronts, and cycle time. For the selected 4-ROI configuration, the absolute deviations in propagation angle and velocity were evaluated for varying wavefront curvatures and apex displacements (Fig. 3g, h). For nearly planar wavefronts (*r* = 1200 px), the angular uncertainty remains below 3°, and the velocity estimation is not noticeably affected by increased apex shifts. However, with increasing curvature, the accuracy becomes more sensitive to the position of the apex relative to the FOV center, which poses a practical limitation for strongly curved wavefronts. In the experimental setup, however, the FOV spans (10 × 7.5) mm^2^ so that spontaneously propagating contraction wavefronts do not exhibit such pronounced curvature, as these would require substantially larger spatial extents. Overall, these results indicate that the measurement approach with the 4-ROI configuration provides sufficient accuracy and robustness for reliable closed-loop operation under experimental conditions, where externally induced motion is minimized, and wavefront curvatures remain moderate compared to the more extreme cases investigated in simulation. At the same time, the 4-ROI enables fast processing with an achievable closed-loop cycle time of 0.26 ms.

### Spatiotemporal closed-loop control of cardiac contraction wavefronts using sparsely sampled data

**Fig. 4:**
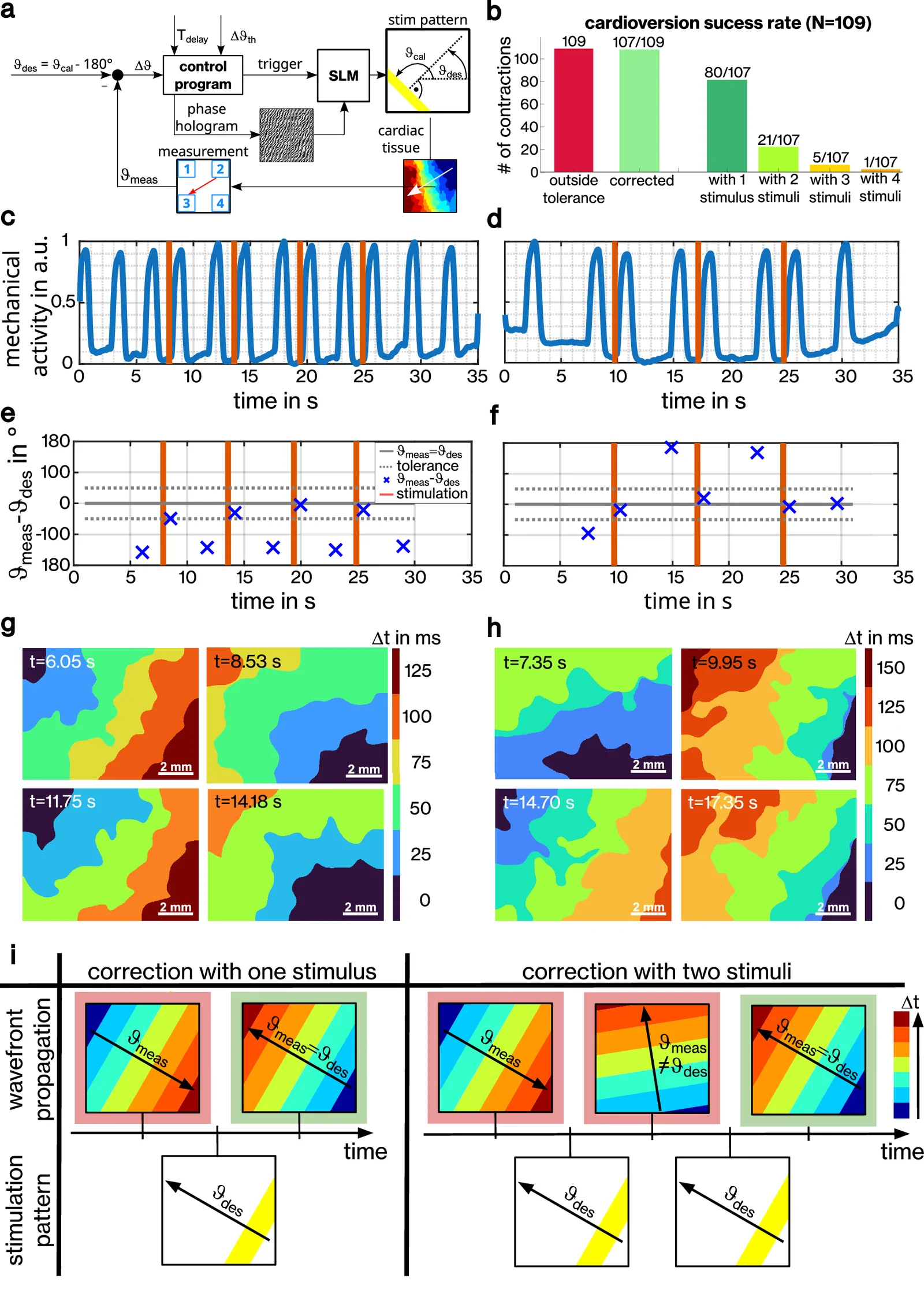
Spatiotemporal closed-loop control of contraction wavefronts. **a** Schematic of the optogenetic closed-loop control of contraction wave propagation. The propagation angle ${\vartheta }_{{{\rm{meas}}}}$ is continuously monitored, and the desired angle ${\vartheta }_{{{\rm{des}}}}$ is chosen as the inverse of the direction ${\vartheta }_{{{\rm{cal}}}}$ measured before the control experiment. If the control error $\triangle \vartheta$ exceeds a predefined threshold ${\triangle \vartheta }_{{{\rm{th}}}}$, a stimulation pattern is applied with a temporal delay ${T}_{{{\rm{delay}}}}$ via the spatial light modulator (SLM), inducing a contraction wave in the desired direction. **b** Number of successful propagation angle corrections over all tries and number of successful corrections after 1,2,3 and 4 stimulations. **c**, **d** Full field mechanical activity with closed-loop-controlled stimulations after a set delay. Orange bars indicate stimulus onset. Stimulation parameters for (**c**, **e**, **g**) were ${t}_{{{\rm{stim}}}}=150\,{{\rm{ms}}},\,{I}_{{{\rm{stim}}}}=171.9\,{{\rm{\mu }}}{{\rm{W}}}/{{{\rm{mm}}}}^{2}$. Stimulation parameters for (**d**, **f**, **h**) were ${t}_{{{\rm{stim}}}}=125\,{{\rm{ms}}},\,{I}_{{{\rm{stim}}}}=381.7\,{{\rm{\mu }}}{{\rm{W}}}/{{{\rm{mm}}}}^{2}$. **e**, **f** Wavefront propagation angle errors for the experiments (**c**, **d**). Blue crosses indicate the measured propagation direction, while orange bars indicate stimulus onsets. Solid gray lines mark a perfect match of the wavefront to the desired angle; dashed gray lines frame the tolerance band which triggers a stimulus when exceeded. **g**, **h** Activity maps showing spontaneous wavefronts (left) and stimulated wavefronts (right). **h** Spontaneous contraction patterns may deviate from their original propagation direction after stimulation. **i** Scheme of optogenetic contraction wavefront correction, requiring either one (left) or two (right) stimuli.

The developed method to measure the characteristics of a planar cardiac contraction wavefront using sparsely sampled signals provides real-time information about spatial properties, such as the propagation angle ϑ. With the known positions of the ROIs, ϑ contains information about the origin of the contraction wavefront within the cellular network. From a clinical perspective, the localization of the excitation origin is relevant for the identification of focal tachycardia, in which activation arises from ectopic sites rather than from the sinoatrial node. Based on this information, stimuli can be applied to restore wavefront propagation in the physiological direction only when the need arises, which opens ways for gentler, energy-efficient cardioversion. In our model system, spontaneous contractions do not arise from a stable origin; instead, their initiation site can vary dynamically, especially under artificial stimulation^45^, reflecting focal-like activation behavior rather than a fixed pacemaker source. Therefore, to demonstrate optogenetic closed-loop control over spatial wavefront properties (control scheme shown in Fig. 4a), we predefined a desired propagation angle ${\vartheta }_{{{\rm{des}}}}={\vartheta }_{{{\rm{cal}}}}-180^\circ$ as a proxy for the physiological propagation direction, where ${\vartheta }_{{{\rm{cal}}}}$ denotes the spontaneous propagation angle determined in a prior experiment. Within the closed loop, the propagation angle for each contraction ${\vartheta }_{{{\rm{meas}}}}$ is measured continuously and in real-time. If the absolute control error $\Delta \vartheta =\left|{\vartheta }_{{{\rm{meas}}}}-{\vartheta }_{{{\rm{des}}}}\right|$ exceeds a predefined threshold ${\Delta \vartheta }_{{{\rm{th}}}}=45^\circ$, an optical stimulus in the above-threshold regime is applied (see Supplementary Note 2), initiating a new contraction wavefront. The stimulation pattern was a light stripe (area: 3.65 mm^2^, width: 0.7 mm) oriented perpendicular to ${\vartheta }_{{{\rm{cal}}}}$ (see Fig. 4a). The stripe was positioned within the FOV; when necessary (e.g., near the corners), it was shifted toward the center while maintaining the orientation to ensure the specified stimulation area was achieved. The optogenetic stimulation was applied with a temporal delay ${T}_{{{\rm{delay}}}}$ after the measurement of the contraction. ${T}_{{{\rm{delay}}}}$ is chosen based on the preceding observation of the spontaneous cardiomyocyte contractions and set with respect to the measured contraction period T to ${T}_{{{\rm{delay}}}}=T/2$. This ensures that the optogenetic stimulus is delivered after the refractory period of the cells but before the onset of the next spontaneous contraction. Representative experiments are visualized in Fig. 4c–h: the left panels illustrate a case where the wavefront direction during spontaneous contractions remained stable over time, whereas the right panels show a case where the direction varied. In Fig. 4c, d, the contraction-derived time signals of the cell cultures are shown. In Fig. 4e, f, the difference ${\vartheta }_{{{\rm{meas}}}}-{\vartheta }_{{{\rm{des}}}}$ between measured and desired propagation angle is plotted over time. Vertical orange lines highlight the time stamps at which the SLM was triggered by the control program. As can be seen in Fig. 4e, f, this triggering occurs when the measured propagation angle exceeds the predefined tolerance range and restores contraction wavefront propagation in the desired direction. Activity maps illustrating representative contraction events are shown in Fig. 4g, h. The experiment was repeated 37 times across nine independent cardiomyocyte cultures, yielding a total of 109 spontaneous contractions outside the defined tolerance range. Of these, 107 contractions (≈98.2 %) were successfully corrected by the applied optical stimulation, as shown in Fig. 4b. In 80/107 cases, a single stimulus was sufficient to achieve correction. If the contraction following optogenetic stimulation did not fall within the tolerance range, a subsequent stimulus was applied with the same temporal delay ${T}_{{{\rm{delay}}}}=T/2$. In these instances, two to four consecutive stimuli were required to establish wavefront propagation in the desired direction. A scheme, exemplarily depicting the case of correction using one optical stimulus and two stimuli is provided in Fig. 4i.

**Fig. 5:**
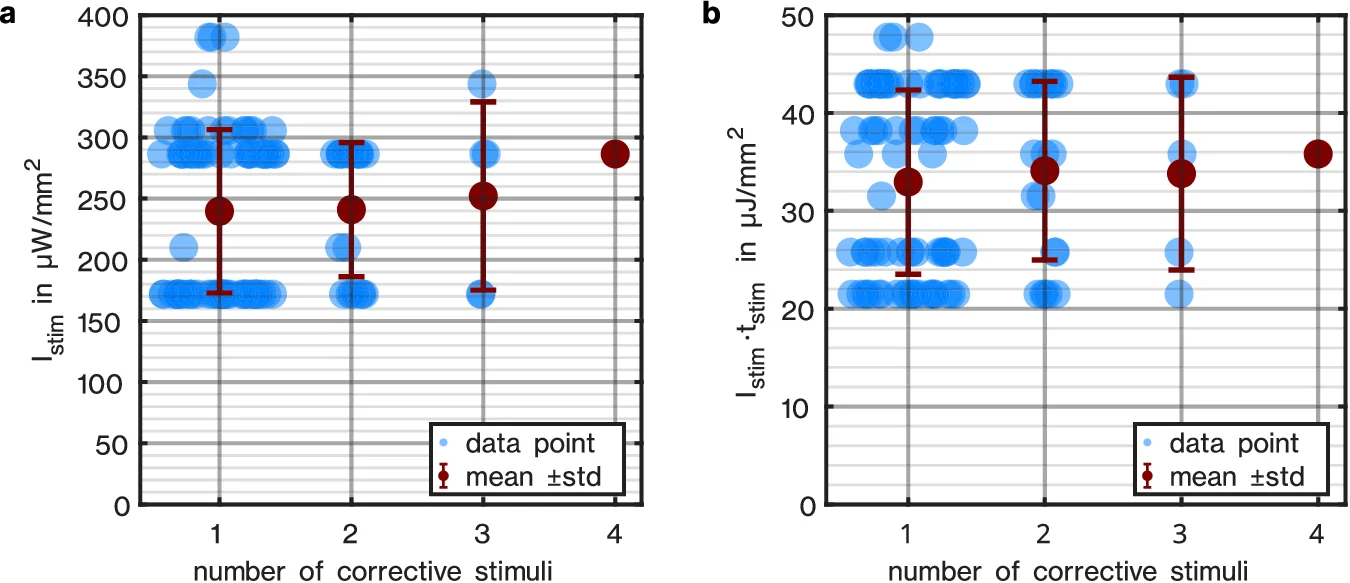
Relationship between number of necessary corrective stimuli and stimulation parameters. Data are presented as mean ± standard deviation. **a** Stimulation intensity ${I}_{{{\rm{stim}}}}$ over number of corrective stimuli. **b** Stimulation fluence ${I}_{{{\rm{stim}}}}\cdot {t}_{{{\rm{stim}}}}$ over number of required stimuli for wavefront correction. Binary logistic regression analysis showed no significant association between the need for multiple corrective stimuli and either stimulation intensity (logistic regression coefficient β=0.001 per ${{\rm{\mu }}}{{\rm{W}}}/{{{\rm{mm}}}}^{2}$, standard error ${{\rm{SE}}}=0.003$ per ${{\rm{\mu }}}{{\rm{W}}}/{{{\rm{mm}}}}^{2}$, odds ratio ${{\rm{OR}}}=1.001$, 95 % confidence interval ${{\rm{CI}}}$ for ${{\rm{OR}}}=[0.995,\,1.008]$, Wald ${\chi }^{2}\left(1\right)=0.180$ and p=0.671) or fluence (β=0.014 per ${{\rm{\mu }}}{{\rm{J}}}/{{{\rm{mm}}}}^{2}$, ${{\rm{SE}}}=0.021$ per ${{\rm{\mu }}}{{\rm{J}}}/{{{\rm{mm}}}}^{2}$, ${{\rm{OR}}}=1.014$, 95 % ${{\rm{CI}}}$ for ${{\rm{OR}}}=[0.972,\,1.058]$, Wald ${\chi }^{2}\left(1\right)=0.442$ andp=0.506). Analysis included 107 spontaneous contraction events from 37 experiments, performed across nine independent cell cultures.

Across all experiments, only two contractions could not be corrected. Notably, in both cases the preceding contractions had already required multiple corrective stimulations, and the uncorrected contraction was always the final event within its respective experiment. This limitation arose because the control loop was terminated after a predefined total experiment duration, reducing the number of corrective stimuli that could be applied by the SLM. Further, the closed-loop control experiments were performed using different light intensity levels (see Fig. 5a).

We assessed a potential relationship between the necessary number of stimuli required for orientation correction and the delivered light intensity using a binary logistic regression model. For this purpose, the outcome was defined as the necessity of more than one corrective stimulus. The analysis revealed no significant effect of light intensity on the probability of requiring multiple stimulations $\left(p=0.671\right)$. Similarly, we examined whether the applied stimulation fluence ${I}_{{{\rm{stim}}}}\cdot {t}_{{{\rm{stim}}}}$ (see Fig. 5b) is associated with the need of more than one corrective stimulus. This analysis likewise showed no significant effect $\left(p=0.506\right)$. Thus, the present data do not provide sufficient evidence to support an association between these stimulation parameters and the need of multiple corrective stimuli.

## Discussion

As the basis for our experiments, we introduced a label-free imaging approach capable of monitoring cardiac contraction wavefront propagation in monolayers through contraction-induced intensity fluctuations in speckle patterns. The motion of the cardiomyocytes is related to the electrical activity via excitation-contraction coupling, with an associated temporal delay^39,40^, whose magnitude and spatial uniformity were not assessed in this study. Under pathological conditions—such as fibrillating cardiac tissue, severe conduction heterogeneity, or impaired calcium handling—this relationship may no longer be preserved. In addition, heterogeneities in the temporal delay between electrical and mechanical activity could limit the ability to infer electrical activity from mechanical motion. In such cases, mechanical activity could still be measured, but its use as a surrogate for electrical activity may be limited, or no longer possible^46^, and derived parameters such as the propagation angle could be biased. However, compared with dye-based techniques, this strategy is advantageous since it avoids phototoxic and cross-sensitivity effects resulting from chemical alteration of the cells^47^. Furthermore, the implementation is straightforward, requiring only an obliquely mounted laser source on a standard microscope. This benefit comes at the cost of higher computational demand for noise filtering, since the method is sensitive to both externally induced and intrinsic motion.

An additional advantage of the label-free approach is the freedom in the selection of the imaging wavelength. This provides compatibility with spectrally diverse optogenetic actuators, enabling simultaneous activation and inhibition^48^ of distinct cardiac regions without spectral crosstalk. Such spectral flexibility could be leveraged for advanced disease-modeling scenarios, such as simulating heterogeneous myocardial environments containing both unexcitable and selectively excitable tissue domains^22,48^. These disease-modeling strategies could be particularly effective in combination with the holographic stimulation system presented here, which achieves single-cell resolution but is at the same time capable of illuminating extended areas as presented in this work to counter source/sink mismatches^49^ preventing true single-cell stimulation. Further, the holographic stimulation concept can be extended to three dimensions using ultrashort-pulse lasers. The application of red-shifted opsins like f-Chrimson as presented in this work allows for longer stimulation wavelengths with reduced scattering effects in tissue^50,51^. Thus, our system lends itself to future investigations in deep-tissue samples such as slices^52,53^. Beyond basic modeling, such holographic approaches provide valuable tools to investigate fundamental cardiac dynamics and to test cardioversion strategies at high spatial precision^26,54^.

While laser-based stimulation is a powerful tool for in vitro investigations, its in vivo translation is limited by the challenge of achieving homogeneous light distribution across the myocardium, especially at depth. Therefore, clinical applications will most likely depend on alternative light-delivery strategies^55–58^. In particular, recent advances in soft materials^55^ show a path to biocompatible implants with reduced foreign body reactions and may provide a promising pathway for future optogenetic pacemakers. Such devices could directly benefit from the sensing approach introduced here.

A major challenge for the realization of closed-loop optogenetic techniques lies in the large data volume required to reconstruct full activity maps of cardiac tissue^44^. In this context, the main innovation of our work is the demonstration of closed-loop optogenetic control over spatial wavefront properties using only a few strategically placed sensors. In our implementation, these sensors were realized optically; however, in principle, any modality capable of capturing local cardiac activity could be applied. Based on signals from a small number of points, the discrimination between planar and rotating contraction waves becomes feasible, and the direction of propagation can be reliably estimated. Sensor positioning does not need to follow a strict geometric arrangement; in principle, a random but known distribution suffices. Nevertheless, deliberate positioning yields advantages, since the detectability of a rotor strongly depends on the relative alignment of regions of interest. A phase delay threshold of 25° was chosen to distinguish planar from rotating wavefronts, based on the maximum phase difference between rising edges of extracted time signals in the investigated hiPSC-CM samples. As described in the results section, this threshold depends not only on the ROI geometry but also on parameters such as the contraction wavefront velocity ${v}_{{{\rm{c}}}}$ and the contraction period T. It should be noted that this threshold is not a universal constant but rather an empirically chosen value determined based on our experimental observations and found to be appropriate across all investigated cases in this study. Theoretically, under conditions where a plane wave satisfies ${v}_{{{\rm{c}}}}\cdot T\le 100\,{{\rm{mm}}}$ (assuming an ROI spacing of 7 mm), this threshold value needs to be adapted, although the underlying principle remains unaffected. Above all, when transferring this approach to a different sample, the concrete value should be adjusted. In situations where beating frequencies reach pathologically high levels and contraction patterns become irregular, deviating from planar wave propagation, the system could still detect such abnormalities based on the local time traces from the ROIs, for example through elevated frequencies or inconsistencies in the extracted contraction periods. In such cases, the stimulation strategy could be adapted accordingly, for instance by applying a global light stimulus to reset the activity of the cardiomyocyte network. Furthermore, the ROI grid could be extended, for example, to a 3 × 3 or 4 × 4 configuration. This could enable a more detailed reconstruction of propagating contraction wavefronts based on angle and velocity measurements in individual subregions, which could help account for fragmented waveforms or identify rotating contraction patterns by comparing the corresponding propagation angles.

Starting from the reduced-data framework, we demonstrated optogenetic closed-loop correction of contraction wavefront orientation. In the present implementation, feedback control was used to adapt stimulation timing based on contractions detected in the ROIs, with the aim of applying stimuli outside refractory periods without requiring prior knowledge of the spontaneous contraction period. Supplementary Fig. S3 shows that pacing efficiency in an open-loop configuration depends on the ratio between stimulation and spontaneous contraction frequency, emphasizing the importance of stimulation timing for successful capture. In addition, calculating the stimulation patterns based on detected wavefront orientation provides a mechanism to account for changes in propagation direction. Together, these aspects motivate the use of feedback-guided stimulation. The developed control algorithm successfully redirected the wavefront in 107 out of 109 cases. The two remaining cases were terminated due to a predefined time cutoff and therefore do not reflect a failure of the control algorithm itself. During our experiments, we observed cases in which multiple consecutive corrective stimuli were required to guide the wavefront in the desired direction. Binary logistic regression analysis did not reveal a significant relationship between the occurrence of multiple corrective stimuli and the applied stimulation parameters, namely stimulation intensity and fluence. Such repetitive optogenetic stimulation may alter local excitability or refractoriness of the cardiomyocytes due to incomplete recovery of ion channels, ionic accumulation, or the finite recovery kinetics of the employed opsin, here f-Chrimson^25,59^. These effects could, in principle, reduce the efficacy of subsequent corrective stimuli and thus represent an interesting direction for future investigations. The corrective stimuli were applied at T/2 after the last observed contraction. This timing assumes sufficient recovery between successive contractions. At high beating frequencies, incomplete diastolic repolarization and mechanical relaxation may lead to reduced excitability, potentially affecting the efficacy and optimal timing of the stimulus. Nevertheless, such high-frequency regimes could be detected by the individual sensors, allowing for the implementation of adapted stimulation strategies to appropriately handle these edge cases. Future studies could evaluate the influence of stimulation delay and angular threshold, set to T/2 and 45°, respectively, in this proof-of-concept study, to optimize correction success, stability, and potentially achieve faster cardioversion. Furthermore, we acknowledge that the present experiments were not performed under faster pacing conditions representative of tachyarrhythmia-relevant frequencies; therefore, future experiments should assess the impact of hyperparameters and performance of the approach under such conditions. As a conclusion, our system demonstrates effective modulation of spatiotemporal wavefront properties without the need for high-density electrode arrays or full-field optical mapping. The implications are considerable: not only does this minimize invasiveness for potential implantable applications, but it also addresses a fundamental bottleneck in energy-efficient rhythm control^44,54^. As outlined in Ref. ^44^, data processing represents the critical limiting factor in ultra-low power scenarios, and we are convinced that the processing scheme proposed here offers a fast and efficient solution.

In a clinical context, reduced-data approaches as presented here may support the discrimination between physiological activation patterns and pathological focal-like activation arising from ectopic sites. Even though our method is currently limited to extracting specific local properties of wavefronts—namely type, velocity, and orientation—it already provides sufficient information for a meaningful characterization of cardiac contractions. Future research could expand this capability to extract further features, such as spiral core position, which is considered essential for optimal optogenetic intervention in arrhythmic behavior^20,28^. In this context, Deng et al. recently demonstrated real-time automatic reentry termination by combining voltage mapping in cardiac monolayers with a machine learning approach^38^. Besides extracting spiral core positions, our proposed wavefront characterization approach will have to be adapted for three-dimensional sample geometries for clinical effectiveness, which could first be investigated in tissue-engineered heart muscle^60^ and cardiac slice cultures^52,53^, which preserve the three-dimensional cellular and fiber architecture and thereby maintain anisotropic conduction behavior. Tissue-engineered heart muscle and precision cut heart slices represent promising intermediate models for such investigations; while tissue-engineered heart muscle represent juvenile myocardium amendable for long term monitoring, manipulation, and design from engineered iPSC models including f-Chrimson iPSC models, precision cut slices can be obtained from patients and transduced to express f-Chrimson using adenovirus-associated virus vectors. Consequently, a clinical translation will require homogenous expression of an optogenetic tool, and further adaptation of the proposed approach based on sparse sampling to appropriately account for anisotropic conduction.

From a technological perspective, the presented system offers high spatiotemporal resolution and great flexibility in stimulation patterns. This makes it ideally suited as a test bed for developing adaptive control schemes that reshape aberrant contraction waves into physiologically relevant patterns, without requiring global full-field stimulation. At present, control frequencies are mainly limited by the camera frame rate of 60 Hz, which is itself constrained by video compression and single-core computer processing power. This limitation affects not only the achievable control cycle time, but also the accuracy of the phase delay estimation, as described by Eq. 2. The relative phase delay error is inversely proportional to the sampling frequency and increases with contraction frequency; therefore, it can become substantial at tachycardia-relevant rates. For example, using representative parameters in Eq. 2 (${v}_{c}=80\,{{\rm{mm}}}/{{\rm{s}}}$ and ${R}_{p,q}\cdot \cos (\vartheta -{\alpha }_{p,q})=7\,{{\rm{mm}}}$), a contraction rate of 250 bpm sampled at 60 Hz yields an estimated relative phase delay error of approximately 52 %, which illustrates the practical limitation of the present camera-based implementation under such conditions. Therefore, to deal with tachycardia rates, the camera could be replaced by a different sensor capable of recording the contraction with a higher temporal resolution. Nevertheless, regardless of the camera, our ROI-based evaluation scheme already supports control operation up to 3.86 kHz on a standard PC, which is enough to deal with tachycardia rates. Further, this could enable subtle modification of incoming contraction waves without triggering de novo excitation. Disregarding the computationally expensive step of hologram generation, further acceleration appears realistic through a field-programmable gate array (FPGA) implementation.

Finally, optogenetic arrhythmia treatment will likely be realized through a combination of biocompatible OLED-array actuators with microelectrode array sensors or similar platforms^55,57,58^. However, we believe that, besides the key issues of biocompatibility, homogeneous expression of an optogenetic tool in the human heart, and implant positioning in the thoracic cavity inside the pericardial space, the decisive factor for clinical translation will not be the light source or sensor alone, but rather the optimal integration of efficient data processing and adaptive stimulation strategies. In this regard, our results demonstrate the feasibility of minimal-data, optogenetic closed-loop control with spatially resolved modulation of cardiac contraction wavefronts, thereby providing a conceptual framework for optical pacemakers that go beyond conventional temporal pacing toward true spatiotemporal rhythm control.

## Methods

The following sections provide a brief overview of the basic procedures of our study; additional explanations are available in the appendix.

### Pluripotent stem cell maintenance

Human induced pluripotent stem cells (hiPSCs; TC1133), originally generated from cord blood-derived CD34+ cells by non-integrating episomal reprogramming as previously described by Baghbaderani et al.^60^, were genetically modified by CRISPR/Cas9-mediated homologous recombination to insert the light-gated ion channel f-Chrimson^36,37^ under the control of the CAG promoter into the AAVS1 safe harbor locus as previously described by Seibertz et al.^61^ Ethical approval has been obtained from the Ethics Committee of the University Medical Center Göttingen (UMG; Ref10/9/15). The cells were maintained under pluripotent conditions in StemMACS™ iPS-Brew XF (Miltenyi Biotec; catalog number 130-104-368). The cells were cultured in T75 tissue culture flasks that were pre-coated with Matrigel (1:120 dilution; 80 µL/cm²) and incubated at 37 °C in a humidified atmosphere containing 5 % CO₂. The medium was refreshed daily, and the cells were passaged at approximately 80 % confluence using EDTA-based dissociation.

### Cardiomyocyte differentiation

The protocol from Tiburcy et al. ^62^ was used. Cardiomyocyte differentiation was initiated when cell confluency reached ≈95 %. On Day 0, the cells were washed with a basal medium consisting of RPMI 1640 (Gibco), supplemented with 1 % penicillin–streptomycin, B27 minus insulin (Gibco #A1895601), 1 mM sodium pyruvate (Gibco #11360088), and 200 µM L-ascorbic acid (Sigma #A8960-5G). To induce mesoderm specification, the same medium was supplemented with 1 µM CHIR99021 (Merck #361559-5MG), 5 ng/mL BMP4 (R&D Systems #314-BP-01M), 5 ng/mL bFGF (Peprotech #AF-100-18B-1000), and 9 ng/mL Activin A (R&D Systems #338-AC-01M) and was applied for 24 hours. Fresh differentiation medium was provided daily until day two. On day three, the cultures were switched to RPMI 1640 containing 1 % penicillin–streptomycin, 2% B27 supplement with insulin (Gibco #17504044), 1 mM sodium pyruvate, 200 µM L-ascorbic acid, and 5 µM IWP4 (ReproCELL #04-0036C). The medium was renewed every other day until day 10.

### Metabolic selection and cardiomyocyte maturation

From day 10 to day 15, metabolic selection was performed by adding glucose-free RPMI 1640, supplemented with 1 % penicillin–streptomycin, 100 µM β-mercaptoethanol, and 440 mM sodium DL-lactate. On day 15, the cells were transferred to a recovery medium consisting of RPMI 1640 supplemented with B27, 1 mM sodium pyruvate, and 200 µM L-ascorbic acid. This medium was replaced every other day. The total number of cells per well (area: 9.6 cm^2^) ranged from 2 to 3 million.

### Experiment protocol

Prior to the optogenetic experiments, cells were supplied with fresh medium and incubated for 1 h. For each experiment, multi-wells were taken out of the incubator and placed in a temperature-controlled humidified stage-top incubator STXF-WSKMX-SET from TOKAI HIT set to 37 °C. For open and closed-loop experiments, spontaneous contraction patterns were recorded to determine spontaneous contraction frequencies and mean contraction pattern directions, followed by the actual experiments.

### Optical setup

The optical setup used throughout this work is depicted shortly in Fig. 1a and detailed in Supplementary Fig. S1a. It consists of an excitation beam path (wavelength: 589 nm) and an observation beam path (wavelength: 660 nm). The excitation beam path is as follows: Collimated light from a fiber-coupled laser is expanded to illuminate a fast binary ferroelectric spatial light modulator displaying computer-generated binary phase-only Fourier holograms. To realize 180° phase modulation, a linear polarizer is mounted in the optical path after the spatial light modulator (SLM)^63^. The holograms are imaged to the back focal plane of the illumination objective with a 4f system of lenses. The holograms’ first diffraction order is selected as the stimulation source of the sample using a rectangular aperture. Illumination patterns on the sample plane are imaged onto a camera using a home-built inverted microscope comprising an objective and a tube lens. Specified light intensities were measured with the power meter PM100D and photodiode S120C, both from Thorlabs, Inc. To track the cardiomyocyte contractions, the samples were further illuminated using a laser source turned into a static speckle pattern using a thin diffuser. Sample-induced fluctuations in speckle intensities are then also imaged onto the camera of the microscope. Since the reliability of speckle-based contraction measurements might decrease in the presence of externally induced motion, such as vibrations from fan-cooled power supplies, all potential sources of vibration were removed from the damped optical table hosting the setup to ensure that changes in speckle pattern intensities are solely attributed to sample motion. Remaining high-frequency artefacts are later suppressed using temporal filters (see Methods sections below and Supplementary Notes 3, 5). To prevent stimulation light from reaching the camera, two notch filters are used. The achieved field of view (FOV) for stimulation is (19 × 9.5) mm^2^. For observation, the FOV is (10 × 7.5) mm^2^. The stimulation based on the SLM provides a refresh rate of 1.7 kHz and an achievable lateral resolution in the single-cell-size range (< 20 µm). Real-time monitoring of contraction wavefronts is possible with up to 3.86 kHz based on our approach, while realizing the sensors optically limits the temporal resolution to 16.67 ms due to the utilized camera.

### Hologram calculation

Computer generated holograms (CGHs) are calculated as outlined in detail in Ref. ^22^. Since the setup is based on Fourier holograms, desired illumination spot positions on the sample are transformed according to Eq. 3a and Eq. 3b,

3a $${P}_{x}={P}_{x}^{{\prime} }\cdot \frac{{N}_{x}d{f}_{{{\rm{tube}}}}}{f\lambda {f}_{{{\rm{fourier}}}}}+\frac{{N}_{x}}{2},$$

3b $${P}_{y}={P}_{y}^{{\prime} }\cdot \frac{{N}_{y}d{f}_{{{\rm{tube}}}}}{f\lambda {f}_{{{\rm{fourier}}}}}+\frac{{N}_{y}}{2},$$

and then Fourier transformed with a random initial phase to match the physical parameters of the spatial light modulator. Here, $[{P}_{x},{P}_{y}]$ is a point on the sample plane, $[{P}_{x}^{{\prime} } ,{P}_{y}^{{\prime} }]$ a coordinate-transformed point in an intermediate image plane before Fourier transforming, ${N}_{x},{N}_{y}$ are the number of pixels in each axis of the SLM, d is the SLM pixel size, f, ${f}_{{{\rm{tube}}}}$ and ${f}_{{{\rm{fourier}}}}$ are the objective’s, tube and Fourier lens focal lengths, and λ the illumination center wavelength. Complex illumination patterns are realized as a superposition of single points with random initial phase. An axial offset Δz with respect to the focal plane of the system was set by superimposing the hologram with a Fresnel lens of the focal length

4 $${f}_{{{\rm{fresnel}}}}=-\frac{{f}^{2}}{\Delta z}\frac{{f}_{{{\rm{fourier}}}}}{{f}_{{{\rm{tube}}}}}\,$$

to axially match the focal plane of the stimulation system with the sample plane. Omitting amplitude information, the phase of the resulting complex-valued holograms is then binarized with a threshold to account for the binary switching behavior of the SLM.

### Detection of contraction events and frequency measurement

To extract a time signal representing the mechanical activity of the cardiomyocytes from speckle patterns, the speckle background was first calculated as the temporal mean over several frames. The absolute intensity difference between each frame and the background was then computed and summed over all pixels of each frame to generate a raw time trace. To reduce high-frequency noise and low-frequency intensity drifts, the signal was band-pass filtered in the frequency domain and subsequently normalized.

### Activity map generation

To obtain spatiotemporal contraction dynamics from intensity fluctuations in the speckle patterns, the average background was first removed from each video frame. The FOV was then divided into equal-sized regions. From each region, a time signal was extracted as described above. To visualize the contraction, not the relaxation, the positive temporal derivatives of all signals were computed. Spatial low-pass filtering, followed by denoising and smoothing operations, lead to visualization of the propagation of contraction wavefronts. To create activity maps, the frames were then binarized and superimposed according to their initial capture time. Remaining noise artefacts were removed by eliminating elements not belonging to the contraction wavefront and by applying spatial low-pass filtering.

### Real-time signal acquisition for sparse sampling

Optogenetic closed-loop control requires the real-time tracing of contraction events at distinct spatial positions. To achieve this, speckle patterns for each region of interest (ROI) were extracted from binned camera images (542 × 720) and compared with a continuously updated, ROI-specific mean background. An ROI-specific time trace was then obtained by summing the absolute differences between the background and the current speckle pattern. This trace was processed in real time by low-pass filtering and normalization to the range $\left[{\mathrm{0,1}}\right]$. The normalized ROI signals were subsequently thresholded at a value of 0.5 to determine contraction times for each ROI and to estimate temporal shifts between contraction events across ROIs.

### Real-time contraction wavefront characterization based on sparsely sampled signals

Based on the temporal shifts in contraction activity between ROIs, plane-wave characteristics such as propagation direction and velocity can be estimated. To implement this in a real-time closed-loop framework, the contraction propagation was approximated as a plane wave. Under this assumption, the spatial distance ${\vec{r}}_{p}-{\vec{r}}_{q}$ and temporal delay $\Delta {t}_{p,q}$ between contractions at two ROIs p and q are related by:

5 $${v}_{c}=\frac{\vec{k}\cdot \left[{\vec{r}}_{p}-{\vec{r}}_{q}\right]}{\left|\vec{k}\right|}\cdot \frac{1}{\Delta {t}_{p,q}}.$$

Here, ${v}_{c}$ denotes the velocity of the wavefront and $\vec{k}$ the wave vector of the plane-wave propagation with the wave vector components ${k}_{x}$ and ${k}_{y}$ from which the propagation angle can be derived: $\tan \left(\vartheta \right)={k}_{y}/{k}_{x}$. To determine the components of the wave vector, a system of equations based on Eq. 5 was constructed. Solving this system requires the positions and temporal delays of at least three ROIs. In our work, four ROIs were used to reduce noise sensitivity and improve robustness, while keeping computational cost low. With our hardware, iteratively solving the overdetermined system originating from four ROIs required less than 500 µs. A simulation to assess limitations of the sparse sensing approach is described in Supplementary Note 7.

### Statistics

Binary logistic regression was used to model the relationship between a continuous predictor (stimulation parameter) and a binary outcome (need for more than one corrective stimuli during the closed-loop experiments). The unit of analysis for the logistic regression was the individual spontaneous contraction event, because both the stimulation parameter and the binary outcome were defined at the event level. Multiple events could originate from the same experiment and cell culture; this repeated-measures structure was therefore reported explicitly by providing the number of events, experiments, and independent cell cultures. No additional random-effect structure was included in this analysis; consequently, the results were interpreted as event-level associations and not as culture-level effects. Inverse-frequency weighting was applied to account for class imbalance. Associations were evaluated using two-sided Wald tests of the logistic regression coefficients, with p<0.05 considered significant. Logistic regression results are reported as the regression coefficient β, its standard error ${{\rm{SE}}}$, the odds ratio ${{\rm{OR}}}$, the 95% confidence interval ${{\rm{CI}}}$ for the odds ratio, the two-sided Wald ${\chi }^{2}(1)$ statistic with 1 degree of freedom, and the corresponding exact p-value. Odds ratios were used as effect-size estimates and were calculated as ${{\rm{OR}}}={e}^{\beta }$. The 95% confidence intervals for the odds ratios were obtained by exponentiating the corresponding 95% confidence intervals of the regression coefficients.

### Reporting summary

Further information on research design is available in the Nature Portfolio Reporting Summary linked to this article.

## Supplementary information

Transparent Peer Review file

Supplementary Information

Reporting Summary

## Data Availability

The data supporting the findings of this study include raw and processed optical recordings, experiment parameters, simulation data, and analysis outputs. These data are currently stored on institutional servers at TUD Dresden University of Technology. Due to the size of the datasets, data are available from the corresponding author, Robert Wendland/robert.wendland@tu-dresden.de, upon reasonable request. Requests will be answered within six weeks, and data will be shared for academic research purposes subject to feasible data transfer and applicable institutional policies.

## Code availability

Pseudocode describing the developed algorithms is provided in the Supplementary Information. The corresponding custom MATLAB scripts used for data analysis, simulations, ROI-based signal extraction, error quantification and plot generation are available from the corresponding author, Robert Wendland (robert.wendland@tu-dresden.de) upon reasonable request. Requests will be answered within six weeks, and code will be shared for academic research purposes subject to applicable institutional policies. The analyses were performed using MATLAB 2023b and 2025a. The specific variables and parameters used to generate and analyze the datasets are provided in the Methods and Supplementary Information.
