# Supplementary material for: All-optical closed-loop control of human cardiomyocyte networks exploiting holographic optogenetics
Source: Commun Eng. 2026 Sep 17;5:159. doi: 10.1038/s44172-026-00779-1 (PMC13586264; doi:10.1038/s44172-026-00779-1)
Supplement: Supplementary file 1 [file 44172_2026_779_MOESM1_ESM.pdf]

nature portfolio

Peer Review File

All-Optical Closed-Loop Control of Human Cardiomyocyte Networks Exploiting Holographic Optogenetics

Corresponding Author: Mr Robert Wendland

This file contains all reviewer reports in order by version, followed by all author rebuttals in order by version.

Version 0:

Reviewer comments:

Reviewer #1

(Remarks to the Author)
This manuscript presents an elegant and technically sophisticated demonstration of spatiotemporal closed-loop control of cardiac wavefront dynamics in human iPSC-derived cardiomyocyte monolayers. By integrating label-free speckle-based imaging with high-speed holographic optogenetic stimulation, the authors establish a minimally invasive, all-optical feedback system capable of detecting and correcting aberrant propagation directions in real time. The primary conceptual contribution lies in the use of sparsely sampled regions of interest (ROIs) to estimate wavefront type, direction, and velocity from phase delays. The authors convincingly demonstrate that planar and rotating waves can be discriminated using a small number of spatially distributed measurement points, and they provide both experimental validation and error analysis to justify the chosen phase threshold. This reduced-data framework is particularly compelling in the context of implantable or energy-constrained systems, where data throughput and processing cost represent major limitations.
Several aspects could be further clarified or expanded.
a) While the study demonstrates sparse ROI–based wavefront estimation and closed-loop control, it lacks systematic evaluation of key hyperparameters. The impact of ROI number, on estimation accuracy, robustness, and latency is not analyzed, leaving the “minimal sensing” claim insufficiently supported. Additionally, the choice of stimulation delay (T/2) and angular threshold (45°) appears empirical, without sensitivity analysis or justification of operating margins. A parametric study examining how these parameters affect correction success, stability, and energy efficiency would significantly strengthen the methodological rigor and generalizability of the proposed framework.
b) The authors should further address the impact of common optical imaging artifacts, particularly imaging noise and motion-related disturbances, on the reliability of the proposed method. Speckle-based approaches are inherently sensitive to global motion, mechanical drift, and vibration, which may introduce false temporal differences unrelated to cardiac contraction. It would be important to clarify how the system distinguishes true contraction-induced speckle fluctuations from external motion artifacts, and whether additional motion compensation, spatial registration, or hardware stabilization was employed. A quantitative robustness analysis under controlled motion perturbations would strengthen confidence in the method’s applicability beyond ideal in vitro conditions.
c) The manuscript would also benefit from a clearer quantitative comparison between the proposed closed-loop strategy and an appropriate open-loop baseline. Although the authors demonstrate high correction success under feedback control, it remains unclear how much improvement is specifically attributable to the closed-loop architecture. For example, would periodic or predefined patterned stimulation (open-loop) achieve comparable directional correction rates? Including controlled experiments comparing correction accuracy, number of stimuli required, recovery time, and robustness to spontaneous variability would more convincingly demonstrate the added value of real-time feedback and substantiate the claim that closed-loop control provides superior spatiotemporal regulation.
Overall, this study represents a significant step toward spatially resolved optogenetic rhythm control. By showing that minimal sensing can suffice for effective closed-loop modulation of wavefront orientation, the authors provide both a conceptual and technological framework that may inform future optical pacemaker or cardioversion strategies. The work will be of interest to researchers in cardiac electrophysiology, optogenetics, and closed-loop control of excitable biological systems.

Reviewer #2

(Remarks to the Author)

I have read the manuscript with interest and would like to raise several points for the authors to clarify. I would appreciate the authors' responses to the following questions.
1. You picked 25° as your threshold for distinguishing planar from rotating waves, based on observing 13 independent cardiomyocyte networks where the maximum measured phase delay stayed below 19.7°. I don't think thirteen is a large sample. What happens if you encounter a culture with unusually slow conduction velocity or a shorter contraction period than anything in your current dataset — couldn't a perfectly planar wave then exceed 25° and get misclassified as rotating? Did you consider making the threshold adaptive on a per-culture basis rather than fixing it as a universal constant?
2. Four ROIs is elegant for simple cases, but what about other fragmented wavefronts, like multiple wavelets, or figure-of-eight reentry — patterns that actually matter in clinical arrhythmia? With only 4 sensors, what does the system output when the plane wave assumption breaks down entirely?
3. Both of the two failures you report occurred at the very end of their respective experiments, where the control loop was terminated before enough corrective stimuli could be applied. So those are not really failures of the control strategy itself — but I think are artifacts of experiment duration. If you had let the loop run longer, would those contractions have been corrected too? I think you should be more careful about how you present this number, because as it stands it conflates the effectiveness of the control scheme with practical constraints in your experiment design. Could you clarify whether there were any genuine cases where the stimulation simply could not redirect the wavefront, independent of timing cutoffs?
4. Your speckle-based approach tracks contraction, which is a mechanical readout downstream of the electrical activation. There is necessarily an electromechanical delay between the action potential wavefront and what you actually measure. May I know how large is this delay, and if it is spatially uniform across the culture? If it varies from region to region, your propagation angle measurements could carry a systematic bias I think. Your manuscript did not show any direct comparison with voltage- or calcium-sensitive dye recordings from the same preparations. In my opinion, such a comparison would go a long way toward validating the fidelity of your approach.
5. You motivate the paper with tachycardia at 100–250 bpm, but your camera runs at 60 Hz. At 250 bpm that gives you roughly 14 frames per cardiac cycle ritgh? But your own Eq. 2 shows the phase delay error scales inversely with sampling rate. So the question is have you estimated the measurement uncertainty at tachycardia rates, and does it stay comfortably below your 25° threshold?
6. In the manuscript, you noted that both uncorrected contractions were preceded by sequences requiring multiple consecutive corrective stimuli. I think this is a pattern that worth exploring. I would like to know does repeated optogenetic pulsing alter local excitability or refractoriness in the tissue? And could there be like opsin desensitization or ionic loading effects that make each subsequent correction progressively harder? If so, that would be a meaningful limitation for any sustained or long-duration application of this approach, and it would be helpful to see some discussion or data on this point.

Reviewer #3

(Remarks to the Author)
In the submitted manuscript entitled 'All-Optical Closed-Loop Control of Networks Exploiting Holographic Optogenetics', Wendland et al. describe the development of a novel experimental setup to assess the possibility of optical steering of propagating electrical waves, with the vision to provide optimised protocols for optogenetic termination of tachyarrhythmia. The proposed system relies on the use of 2D monolayers of hiPSC-derived cardiomyocytes expressing the red-light-gated cation channel Chrimson, allowing them to precisely trigger membrane depolarisation in the illuminated region. There are two main outcomes/achievements from the study: (1) Propagating electrical waves can be estimated from cell deformations, i.e. contractions, and Wendland et al. showed how sparse spatial sampling is sufficient to deduce the deviation of the direction of conduction from 'normal' conduction. (2) A line ('strip') of light applied during the 'diastolic' phase and perpendicular to the propagating wavefront is sufficient to restore normal conduction patterns. The provided technology, especially the implementation of sparse sampling, is compelling, providing a step towards closed-loop control of cardiac activity. However, the feasibility of the proposed approach in 3D tissue, as well as the underlying mechanisms of wavefront steering, remain to be still elucidated.
Please find my suggestions for improving the manuscript below:
- Introduction, but also in the discussion: Please carefully distinguish between electrical activation/deactivation and contraction/relaxation throughout the study, especially in the introduction. While these two processes are physiologically coupled via excitation-contraction coupling, they may not be coupled in the case of tachyarrhythmia, and such coupling might be absent in fibrillating tissue. Please revise the entire introduction accordingly, and explain in which cases deformation can be used as a surrogate for electrical activation (of course, with a temporal delay). Please also revise terminology, for example 'contraction rhythm' is not a commonly used term in the field.
- The distinction between focal tachycardia and reentry tachycardia is unclear given the scope of the study. In the introduction, you may want to distinguish between atrial and ventricular arrhythmia, given that contraction of the heart is mainly driven by the ventricular walls. I would advise to first explain the different electrical activation patterns, before discussing the resulting alterations in (ventricular) contraction-relaxation behaviour. Please also adjust passages in the results part/discussion, e.g. lines 234-36 and lines 338-340. In case of a not sinus-node driven atrial tachyarrhythmia, ventricular activation maps might still appear normal, as ventricular activation would be AV-node driven. Depending on the atrial rhythm, there might be a partial or even full dissociation between atrial and ventricular activation.
- Throughout the study, the authors claim to have implemented the first all-optical, closed-loop strategy for cardiac arrhythmia termination. However, closed-loop optogenetic approaches have been suggested more than ten years ago (e.g. see https://pmc.ncbi.nlm.nih.gov/articles/PMC4775736/) and have been successfully applied to cardiac tissue or cells (e.g. see https://pmc.ncbi.nlm.nih.gov/articles/PMC10753386/; and very recently: https://pubmed.ncbi.nlm.nih.gov/41684280/; https://www.science.org/doi/10.1126/sciadv.abq7469)
- Please also refer to the pioneering work by Jan Christoph, demonstrating the feasibility of using mechanical activity to infer electrical activity, e.g. https://pubmed.ncbi.nlm.nih.gov/33380038/

- The electrical and mechanical connectivity of hiPSC-cardiomyocytes strongly depend on the density of the cells in the culture. Please provide the number of cells seeded by area, and please provide a representative image of the cells within the measurement chamber.
- You choose a phase delay of 25° to distinguish a planar wave from a rotating wave. How translatable is this distinction to cardiac tissue with physiologically occurring conduction pathways and their variations? Why did you use 45° as threshold for applying light later on?
- If I understand correctly, the light stimulus is delivered in-between two contractions (e.g. at T/2). Please discuss limitations of this approach, especially in the case of fast rhythms when full diastolic repolarisation/relaxation might not be reached.
- In addition, you apply a light strip perpendicular to the desired wavefront propagation to reset the direction of the wavefront. In Figure 3, you state two different light intensities and two different durations of light applied. How did you determine the optimal parameters? Why did they differ between individual experiments? Which wavelength was used? What were the x-y dimensions of the applied light strip, and did it always extend to the boundaries of the field of view/chamber? To further understand the methodology, it would be beneficial to include a time-resolved schematic with a propagating wavefront and the corresponding illumination pattern(s), both depicting a case with single illumination and one where multiple attempts were needed.
- Please explain the underlying mechanism by which the wavefront was re-directed. Did you test different light intensities (e.g. sub-threshold and above-threshold regimes)?
- In Figure 2, the provided conduction velocities are significantly lower than in normal myocardium, and the provided rates are unphysiologically low. Have you tested the presented cardioversion approach at frequencies as present in tachyarrhythmia e.g. at 2 Hz or faster?
- Please discuss challenges of translating the presented cardioversion approach into 3D tissue and myocardium. In this regard you mention the use of 3D organoids. How representative are currently available organoid models in respect to anisotropic conduction velocity as present in normal myocardium? Why not use tissue slices instead?
- Please make sure to label/explain all variables used in the Figures in the Figure legends. In Figure 2f-h please label the x-axes of the graphs provided.
- Please correct spelling mistakes/typos.

Version 1:

Reviewer comments:

Reviewer #1

(Remarks to the Author)
The authors have made a substantial effort to address the reviewers' concerns. In particular, the added simulation-based analysis of ROI number, ROI geometry, noise sensitivity, wavefront curvature, and achievable cycle time strengthens the methodological basis of the sparse-sensing framework.However, one concern remains insufficiently resolved.The added analyses do not fully address the need for an appropriate open-loop baseline. The rebuttal explains why open-loop stimulation is expected to be less robust, but this is not equivalent to a controlled comparison using the same directional-correction endpoint. The manuscript should either include a matched open-loop comparison, even if limited, or substantially soften claims that the closed-loop strategy provides superior spatiotemporal regulation.In summary, the revision improves the manuscript, I would recommend a minor revision before acceptance.

Reviewer #2

(Remarks to the Author)
Thank you for the thorough revision. Most concerns have been satisfactorily addressed.

Two minor textual additions I think would help the final version: (1) an explicit acknowledgement that the spatial uniformity of the electromechanical delay was not measured, and that spatially heterogeneous delay could bias propagation angle estimates; and (2) inclusion of the quantitative phase delay error at tachycardia rates (~52% at 250 bpm) discussed in the rebuttal but absent from the manuscript.

I do not anticipate a further round of review.

Reviewer #3

(Remarks to the Author)
The authors have carefully revised the manuscript, addressing all points raised during the initial review.

Version 2:

Reviewer comments:

Reviewer #1

(Remarks to the Author)

The authors have carefully revised the manuscript, addressing all points raised during the initial review.

Open Access This Peer Review File is licensed under a Creative Commons Attribution 4.0 International License, which permits use, sharing, adaptation, distribution and reproduction in any medium or format, as long as you give appropriate credit to the original author(s) and the source, provide a link to the Creative Commons license, and indicate if changes were made.
In cases where reviewers are anonymous, credit should be given to 'Anonymous Referee' and the source.
The images or other third party material in this Peer Review File are included in the article's Creative Commons license, unless indicated otherwise in a credit line to the material. If material is not included in the article's Creative Commons license and your intended use is not permitted by statutory regulation or exceeds the permitted use, you will need to obtain permission directly from the copyright holder.
To view a copy of this license, visit https://creativecommons.org/licenses/by/4.0/

Response to the Reviewers

Nature Communications Engineering submission,
Manuscript ID: COMMSENG-26-0039,
Type: research article

Title: All-Optical Closed-Loop Control of Human Cardiomyocyte Networks Exploiting Holographic Optogenetics

Authors: Robert Wendland, Felix Schmieder, Muhammad Atif Sikandar, Frithjof Paul Knüppel, Wolfram-Hubertus Zimmermann, Olaf Bergmann, Lars Büttner, Jürgen Czarske

black: Comments of the Reviewers
blue: the authors´ reply to these comments

Please note that the referenced text passages refer to the revised manuscript (**with markup**).

Reviewer 1:

1. While the study demonstrates sparse ROI–based wavefront estimation and closed-loop control, it lacks systematic evaluation of key hyperparameters. The impact of ROI number, on estimation accuracy, robustness, and latency is not analyzed, leaving the "minimal sensing" claim insufficiently supported. Additionally, the choice of stimulation delay (T/2) and angular threshold (45°) appears empirical, without sensitivity analysis or justification of operating margins. A parametric study examining how these parameters affect correction success, stability, and energy efficiency would significantly strengthen the methodological rigor and generalizability of the proposed framework.

To strengthen the methodological evaluation of the proposed sparse sensing approach, we added a simulation-based study investigating the impact of key hyperparameters of this framework. Specifically, we evaluated the influence of the number/ geometry of ROIs on angle and velocity measurement accuracy, robustness against noise and the impact of deviations from ideal planar wave propagation. We further added achievable cycle times/ latencies for different ROI numbers. These results are presented in section 2.3 and figure 3 of the revised manuscript.
Regarding the choice of stimulation delay and the angular threshold, we agree that a systematic optimization and sensitivity analysis of these parameters would be highly valuable. In the present proof-of-concept study, these parameters were selected empirically to demonstrate the feasibility of the closed-loop framework. We therefore clarified the rationale underlying the selection of these and mentioned their systematic evaluation and optimization for future work in the discussion (see lines 519-521, 525-527).
However, we analyzed the relationship between stimulation parameters (intensity and fluence) and the necessity of multiple corrective stimuli and added it to the manuscript (see section 2.4 and figure 5).

2. The authors should further address the impact of common optical imaging artifacts, particularly imaging noise and motion-related disturbances, on the reliability of the proposed method. Specklebased approaches are inherently sensitive to global motion, mechanical drift, and vibration, which may introduce false temporal differences unrelated to cardiac contraction. It would be important to clarify how the system distinguishes true contraction-induced speckle fluctuations from external motion artifacts, and whether additional motion compensation, spatial registration, or hardware stabilization was employed. A quantitative robustness analysis under controlled motion

perturbations would strengthen confidence in the method's applicability beyond ideal in vitro conditions.

Since speckle-based approaches are inherently sensitive to externally induced motion, the experimental setup was mounted on a damped optical table and potential vibration sources such as fan-cooled power supplies were removed from the table to minimize these artifacts. In addition, remaining high-frequency disturbances and slow drift components were suppressed by temporal filtering within the evaluation algorithms. These hardware- and software-based stabilization strategies are now described (with references to respective supplementary information) in the revised Methods section 4.5.
Further, we agree that controlled experimental perturbation studies would provide additional insight into the robustness of the method. However, such experiments were considered beyond the scope of the study, as the proposed speckle-based imaging approach is intended for *in vitro* applications, where environmental disturbances can generally be minimized effectively. Nevertheless, as mentioned in the response to comment 1, we incorporated a simulation-based analysis of noise sensitivity. We integrated a simulation-based study on the influence of noise as mentioned in the response to 1.). The simulated noise was modeled as random phase perturbations in the sample plane, representing high-frequency externally induced motion artifacts (a full methodological description of the simulation can be found in Supplementary Note 7).

3. The manuscript would also benefit from a clearer quantitative comparison between the proposed closed-loop strategy and an appropriate open-loop baseline. Although the authors demonstrate high correction success under feedback control, it remains unclear how much improvement is specifically attributable to the closed-loop architecture. For example, would periodic or predefined patterned stimulation (open-loop) achieve comparable directional correction rates? Including controlled experiments comparing correction accuracy, number of stimuli required, recovery time, and robustness to spontaneous variability would more convincingly demonstrate the added value of real-time feedback and substantiate the claim that closed-loop control provides superior spatiotemporal regulation.

A comparison to an open-loop strategy is essential to highlight the benefit of feedback control. In an open-loop configuration, successful correction/ pacing depends on prior knowledge of contraction parameters, in particular the period of spontaneous contraction and time relative to the refractory state of the cell network. This claim is supported by the data presented in Supplementary Fig. S3, which shows that successful pacing and capture strongly depends on the ratio between stimulation and spontaneous contraction frequency. The proposed closed-loop approach does not require a priori knowledge of these temporal dynamics and can adapt stimulation-timing-based feedback about contractions in the ROIs, thereby ensuring stimulation outside refractory periods. Moreover, open-loop strategies inherently lack the ability to adapt to spatially varying propagation characteristics, such as changes in wavefront direction.
We have clarified this comparison in the revised manuscript and further emphasized the role of feedback control (see lines 502-509).

Reviewer 2:

1. You picked 25° as your threshold for distinguishing planar from rotating waves, based on observing 13 independent cardiomyocyte networks where the maximum measured phase delay stayed below 19.7°. I don't think thirteen is a large sample. What happens if you encounter a culture with unusually slow conduction velocity or a shorter contraction period than anything in your current dataset —couldn't a perfectly planar wave then exceed 25° and get misclassified as rotating? Did you

consider making the threshold adaptive on a per-culture basis rather than fixing it as a universal constant?

We fully agree, that the chosen threshold should not be understood as a universal constant. This value depends on parameters like wavefront velocity, contraction period and ROI geometry. This has been additionally clarified in the discussion of the revised manuscript (see lines 480-486). In particular, we emphasized that the 25° threshold was selected based on experimentally observed cardiomyocyte networks and is therefore appropriate for the investigated conditions, rather than intended as a general fixed value.
Importantly, this threshold is already partially adaptive, as phase delays which are compared to the threshold values are calculated relative to the last observed culture-specific contraction period (see Eq. 1).
Furthermore, as noted by the reviewer, in extreme cases with slow velocity v_c or a short contraction period T (compared to the last observed one), a perfectly planar wave could in principle exceed this threshold. Within our ROI configuration (assuming an ROI spacing of 7 mm), such cases would correspond to $v_c \cdot T \leq 100\ \mathrm{mm}$ and were not observed within our dataset. This consideration is included in the Discussion of the revised manuscript (see lines 486-490).

2. Four ROIs is elegant for simple cases, but what about other fragmented wavefronts, like multiple wavelets, or figure-of-eight reentry — patterns that actually matter in clinical arrhythmia? With only 4 sensors, what does the system output when the plane wave assumption breaks down entirely?

We agree that more complex contraction patterns represent clinically highly relevant scenarios that are not fully captured by the present 4-ROI implementation. The current approach was designed for characterization of predominantly planar and rotating patterns. In principle, the sparse sensing approach can be expanded to larger ROI arrays (e.g., 3×3 or 4×4 configurations), enabling estimation of propagation angle and velocity within spatial subregions and thereby facilitating reconstruction of more fragmented or spatially heterogeneous wave dynamics. We included this potential modification in the Discussion (see lines 495-499).
When the plane wave assumption breaks down, the 4 ROI configuration remains capable of identifying abnormalities through inconsistencies, such as spatially varying contraction periods or in general elevated contraction frequencies. Spiral wave propagation can be distinguished based on the phase delays as described in section 2.2. In such cases, alternative stimulation strategies could be triggered, for example global reset stimuli. This is now also mentioned in the Discussion of the revised manuscript (see lines 490-495).
In addition, to better characterize the limitations of the plane-wave assumption, we performed a simulation-based analysis investigating the influence of wavefront curvature and apex displacement on the uncertainty of propagation angle and velocity estimation (section 2.3 and figure 3 in the revised manuscript).

3. Both of the two failures you report occurred at the very end of their respective experiments, where the control loop was terminated before enough corrective stimuli could be applied. So those are not really failures of the control strategy itself — but I think are artifacts of experiment duration. If you had let the loop run longer, would those contractions have been corrected too? I think you should be more careful about how you present this number, because as it stands it conflates the effectiveness of the control scheme with practical constraints in your experiment design. Could you clarify whether there were any genuine cases where the stimulation simply could not redirect the wavefront, independent of timing cutoffs?

We thank the reviewer for this helpful remark, to which we totally agree. The two instances of non-correction occurred at the very end of their respective experiments, where the control loop had already been terminated and no further corrective stimuli could be applied. To avoid conflating these

timing-related limitations with the performance of the control strategies, we have clarified in the Discussion that the two cases do not reflect a failure of the control algorithm itself (see lines 509-511) and added “at least” in the abstract where we presented the number of correction success.

4. Your speckle-based approach tracks contraction, which is a mechanical readout downstream of the electrical activation. There is necessarily an electromechanical delay between the action potential wavefront and what you actually measure. May I know how large is this delay, and if it is spatially uniform across the culture? If it varies from region to region, your propagation angle measurements could carry a systematic bias I think. Your manuscript did not show any direct comparison with voltage- or calcium-sensitive dye recordings from the same preparations. In my opinion, such a comparison would go a long way toward validating the fidelity of your approach.

We agree that our speckle-based approach measures contraction-associated mechanical deformation downstream of electrical activation, rather than membrane voltage or calcium directly. We have clarified in Section 2.1 that this readout can serve as a surrogate for electrical activation when excitation–contraction coupling is preserved (see lines 108-111). Previous studies support this concept. Christoph et al. (doi: 10.1063/5.0023751) and Liu et al. (doi: 10.1021/acsphotonics.2c01644) demonstrated that activation dynamics can be inferred from mechanical motion under controlled conditions. In particular, Liu et al. showed comparable wavefront velocities and similar spatial patterns when using electrical and mechanical readouts in cardiac monolayers. We have included these references as suggested by Reviewer 3. Based on these references, the electromechanical delay is on the order of <300 ms, but its magnitude and spatial uniformity were not measured. We now include the possibility of differences between electrical excitation and mechanical activity based on spatial heterogeneity (see lines 437-439). In addition, we included lines 434-441 in the Discussion addressing that in case of fibrillating tissue, severe conduction heterogeneity or impaired calcium handling, the excitation-contraction coupling may be no longer valid.

5. You motivate the paper with tachycardia at 100–250 bpm, but your camera runs at 60 Hz. At 250 bpm that gives you roughly 14 frames per cardiac cycle ritgh? But your own Eq. 2 shows the phase delay error scales inversely with sampling rate. So the question is have you estimated the measurement uncertainty at tachycardia rates, and does it stay comfortably below your 25° threshold?

We agree that at tachycardia rates, the phase-delay error becomes a practical limitation, when the camera caps the measurement frequency at its framerate of 60 fps. For example, using exemplary values of $v_c = 80\ \mathrm{mm/s}$, $R_{p,q} \cdot \cos(\vartheta - \alpha_{p,q}) = 7\ \mathrm{mm}$ and $1/T = 250\ \mathrm{bpm}$, Eq. 2 yields a relative error of approx. 52 %. We now mention the framerate-related limitation in the Discussion (see lines 566-571). We further clarified that, in our implementation, the computation can run at up to 3.86 kHz. Thus, if the camera is replaced by a sensor with higher sampling rate, the relative error of phase delay measurement would decrease to approx. 0.8 %. This indicates that the limitation arises primarily from the imaging frame rate rather than from the computation algorithm itself.
Regarding the 25° threshold, we clarified that this value was suitable for our observations but should not be interpreted as a universal constant (see lines 480-490). For example, for a ROI spacing of 7 mm, a plane wave satisfying $v_c \cdot T \leq 100\ \mathrm{mm}$ could be misclassified. Therefore, particularly when investigating different samples or using a different ROI geometry, this threshold needs to be adapted accordingly.

6. In the manuscript, you noted that both uncorrected contractions were preceded by sequences requiring multiple consecutive corrective stimuli. I think this is a pattern that worth exploring. I would like to know does repeated optogenetic pulsing alter local excitability or refractoriness in the tissue? And could there be like opsin desensitization or ionic loading effects that make each subsequent correction progressively harder? If so, that would be a meaningful limitation for any sustained or longduration application of this approach, and it would be helpful to see some discussion or data on this point.

The occurrence of uncorrected contractions after sequences requiring multiple consecutive corrective stimuli is an important pattern and may indicate a relevant limitation for sustained or long-duration applications. We have therefore expanded the Discussion to address the possibility that repetitive optogenetic stimulation could alter local excitability or refractoriness of the cardiomyocytes, for example due to incomplete recovery of ion channels, ionic accumulation, or the finite recovery kinetics of the employed opsin (see lines 515-519). Such effects could, in principle, reduce the efficacy of subsequent corrective stimuli and make repeated corrections progressively more difficult.
In addition, we investigated whether different stimulation intensities/fluences affected the need for multiple corrective stimuli. The corresponding results have been added to Section 2.4.

Reviewer 3:

1. Introduction, but also in the discussion: Please carefully distinguish between electrical activation/deactivation and contraction/relaxation throughout the study, especially in the introduction. While these two processes are physiologically coupled via excitation-contraction coupling, they may not be coupled in the case of tachyarrhythmia, and such coupling might be absent in fibrillating tissue. Please revise the entire introduction accordingly, and explain in which cases deformation can be used as a surrogate for electrical activation (of course, with a temporal delay). Please also revise terminology, for example 'contraction rhythm' is not a commonly used term in the field.

We thank the reviewer for this important comment. We agree that electrical activation/deactivation and mechanical contraction/relaxation should be clearly distinguished throughout the manuscript. Although these processes are linked through excitation–contraction coupling, where electrical activation precedes and initiates mechanical contraction, this relationship may not always be preserved, particularly in tachyarrhythmic or fibrillating tissue. We have revised the Introduction and Section 2.1 to state more explicitly that our approach uses mechanical motion as surrogate for electrical activation, with an associated temporal delay (lines 82-84 and 108-111). In the Discussion, we now additionally address the limitation that, for example in fibrillating tissue, the coupling between electrical activation and mechanical deformation may not be guaranteed, which limits the applicability of deformation-based inference of electrical activation (see lines 434-441).
We also revised the terminology throughout the manuscript to consistently distinguish between electrical activity and mechanical motion/ contraction, and replaced non-standard wording such as "contraction rhythm".

2. The distinction between focal tachycardia and reentry tachycardia is unclear given the scope of the study. In the introduction, you may want to distinguish between atrial and ventricular arrhythmia, given that contraction of the heart is mainly driven by the ventricular walls. I would advise to first explain the different electrical activation patterns, before discussing the resulting alterations in (ventricular) contraction-relaxation behaviour. Please also adjust passages in the results part/discussion, e.g. lines 234-36 and lines 338-340. In case of a not sinus-node driven atrial tachyarrhythmia, ventricular activation maps might still appear normal, as ventricular activation would be AV-node driven. Depending on the atrial rhythm, there might be a partial or even full dissociation between atrial and ventricular activation.

We revised the Introduction to more clearly distinguish between focal and reentrant activation patterns, as well as between atrial and ventricular arrhythmias. In particular, we now first describe the underlying electrical activation patterns before discussing their possible consequences for contraction. We also clarified that our study focuses on local activation dynamics in simplified *in vitro* monolayers, while whole-heart atrial and ventricular activation can be partially or fully decoupled due to AV-node

conduction (see lines 51-59). Furthermore, we now explicitly state that mechanical deformation is used as a surrogate for electrical activation based on excitation–contraction coupling, while the associated temporal delay and the limitations of this surrogate relationship are integrated in Section 2.1 (see lines 108-111) and in the Discussion (see lines 434-441). We also adjusted the mentioned passages in the Results part and the Discussion (see lines 347-350 and 539-542).

3. Throughout the study, the authors claim to have implemented the first all-optical, closed-loop strategy for cardiac arrhythmia termination. However, closed-loop optogenetic approaches have been suggested more than ten years ago (e.g. see https://pmc.ncbi.nlm.nih.gov/articles/PMC4775736/) and have been successfully applied to cardiac tissue or cells (e.g. see https://pmc.ncbi.nlm.nih.gov/articles/PMC10753386/; and very recently: https://pubmed.ncbi.nlm.nih.gov/41684280/; https://www.science.org/doi/10.1126/sciadv.abq7469)

We agree that closed-loop optogenetic approaches have previously been proposed and applied in cardiac research, including real-time all-optical stimulation strategies for controlling cardiac rhythm and closed-loop all-optical cardiac electrophysiology approaches. We have therefore revised the manuscript to avoid overstating the novelty of our work. Specifically, we now mention the concept of all-optical closed-loop strategies in the Introduction and have integrated the relevant literature suggested by the reviewer (see lines 71-73). In addition, we added the recent all-optical closed-loop approach for spiral wave termination by Deng *et al*. in the Discussion (see lines 546-548). To avoid any ambiguity, we have also removed the phrase "for the first time" throughout the manuscript. The focus of our work is the implementation of closed-loop control not only in the temporal domain, i.e., stimulus timing, but also in the spatial domain, i.e., feedback-based adaptation of the illumination pattern according to the detected wavefront orientation. This spatial feedback component enables the system to respond to ongoing changes in propagation direction and thereby extends beyond closed-loop strategies based solely on temporal triggering.

4. Please also refer to the pioneering work by Jan Christoph, demonstrating the feasibility of using mechanical activity to infer electrical activity, e.g. https://pubmed.ncbi.nlm.nih.gov/33380038/

Thanks for this hint. We have now included the reference by Christoph and Lebert when describing the surrogate approach in section 2.1 (see line 110), namely the use of mechanical activity to infer electrical activity.

5. The electrical and mechanical connectivity of hiPSC-cardiomyocytes strongly depend on the density of the cells in the culture. Please provide the number of cells seeded by area, and please provide a representative image of the cells within the measurement chamber.

Since the electrical and mechanical connectivity of hiPSC-CMs depends on cell density, we have added the seeding density to the Methods section (see lines 613-614). Specifically, 2-3 million cells were seeded per well (area: 9.6 cm^2).
We have included a representative image of the cells within the measurement chamber in Fig S1 of the Supplementary Information and mentioned the name of the utilized incubator in the Methods section 4.4.

6. You choose a phase delay of 25° to distinguish a planar wave from a rotating wave. How translatable is this distinction to cardiac tissue with physiologically occurring conduction pathways and their variations? Why did you use 45° as threshold for applying light later on?

The 25° phase-delay threshold should not be interpreted as a universal constant. As mentioned in the respective Results section (see lines 223-227) and now additionally clarified in the Discussion (see lines 480-490), this threshold is suitable for the local observations and ROI geometry used in the present

implementation, but it depends on parameters such as ROI spacing, wavefront velocity, and contraction period. Therefore, when applying the approach to different samples, different geometries, or tissue with physiologically occurring conduction pathways and greater heterogeneity, the threshold would need to be adapted accordingly.
In more complex propagation patterns, the current sparse sampling strategy based on a 2×2 ROI arrangement may need to be extended, for example to a 3×3 or 4×4 grid. Such an extension would allow a more detailed reconstruction of the local propagation direction and could also support rotor identification by comparing propagation angles across multiple local regions. We now mention this as a possible direction for future studies (see lines 495-499).
We would like to clarify the distinction between the two thresholds, as both are expressed in degrees but refer to different quantities. The 25° threshold refers to a phase-delay threshold used to distinguish between planar and rotating contraction wavefronts. In contrast, the 45° threshold used later for light application is an angular threshold related to the spatial propagation direction of the wavefront. This threshold was chosen because the goal of the closed-loop intervention is to control spatial wavefront properties — specifically, the propagation direction — in a proof-of-concept framework.

7. If I understand correctly, the light stimulus is delivered in-between two contractions (e.g. at T/2). Please discuss limitations of this approach, especially in the case of fast rhythms when full diastolic repolarisation/relaxation might not be reached.

Applying the corrective stimulus at $T/2$ after the last observed contraction assumes sufficient recovery between successive contractions. We have now added a discussion of this limitation (see lines 519-523). In particular, at high beating frequencies, full diastolic repolarization and mechanical relaxation may not be reached before the next stimulus, which could reduce local excitability and thereby affect both the efficacy and the optimal timing of the corrective stimulation. We further note in the Discussion that such high-frequency regimes could be detected by the individual sensors in our closed-loop framework (see lines 523-525). This would allow future implementations to adapt the stimulation timing or strategy accordingly, for example to better handle cases in which incomplete recovery between contractions limits the effectiveness of stimulation.

8. In addition, you apply a light strip perpendicular to the desired wavefront propagation to reset the direction of the wavefront. In Figure 3, you state two different light intensities and two different durations of light applied. How did you determine the optimal parameters? Why did they differ between individual experiments? Which wavelength was used? What were the x-y dimensions of the applied light strip, and did it always extend to the boundaries of the field of view/chamber? To further understand the methodology, it would be beneficial to include a time-resolved schematic with a propagating wavefront and the corresponding illumination pattern(s), both depicting a case with single illumination and one where multiple attempts were needed.

The stimulation was applied in the above-threshold regime. The corresponding strength-duration curve is provided in Supplementary Note 2. Different stimulation durations and intensities were used across the closed-loop experiments. To assess whether these stimulation parameters influenced the number of required corrective stimuli, we have now added an evaluation of this relationship in section 2.4.
The wavelength used for optogenetic stimulation throughout the manuscript was 589 nm, corresponding to the action spectrum peak of Chrimson. This was already stated in section 2.1 (see lines 96-97) and now added to the Methods section (see lines 624 and 625).
Further, we have added a paragraph in section 2.4 describing the geometry of the applied light stripe (see lines 363-366). The light stripe had an area of 3.65 mm^2 and always extended to the boundaries of the FOV.
Following the reviewer's suggestion, we added a time-resolved schematic illustrating the relationship between the propagating wavefront and the corresponding illumination pattern. This schematic is now

included in Figure 4 and depicts a case in which a single corrective stimulus was sufficient and a case in which two corrective stimuli were required.

9. Please explain the underlying mechanism by which the wavefront was re-directed. Did you test different light intensities (e.g. sub-threshold and above-threshold regimes)?

We have clarified in section 2.4 that wavefront redirection was achieved using above-threshold optogenetic stimulation, initiating a new contraction wavefront (see lines 360-362).
The stimulation parameters were chosen in the above-threshold regime to ensure reliable activation, as supported by the strength-duration curve provided in Supplementary Note 2. We tested different light intensities within this above-threshold regime and, as mentioned in our response to the previous comment, we now included in section 2.4 an evaluation of the impact of stimulation intensity/ fluence on the need for multiple corrective stimuli.

10. In Figure 2, the provided conduction velocities are significantly lower than in normal myocardium, and the provided rates are unphysiologically low. Have you tested the presented cardioversion approach at frequencies as present in tachyarrhythmia e.g. at 2 Hz or faster?

We thank the reviewer for raising this point. No, the presented cardioversion approach was not tested at frequencies of 2 Hz or higher in the current study. The experiments were conducted at the spontaneous beating rates of the hiPSC-CM monolayers, which were lower than typical tachyarrhythmic rates, and the conduction velocities were also lower than those observed in normal myocardium. We now acknowledge this as a limitation of the present proof-of-concept study and clarify that future experiments should assess the performance of the approach under faster pacing conditions that better reflect tachyarrhythmia-relevant frequencies (see lines 527-530). However, we consider the challenges associated with higher frequencies to be primarily technical rather than conceptual, since the approach could in principle be extended to faster regimes using components with sufficiently high temporal resolution, including faster sensors.

11. Please discuss challenges of translating the presented cardioversion approach into 3D tissue and myocardium. In this regard you mention the use of 3D organoids. How representative are currently available organoid models in respect to anisotropic conduction velocity as present in normal myocardium? Why not use tissue slices instead?

We thank the reviewer for this important comment. We agree that translation of the presented approach from monolayers to 3D tissue and ultimately myocardium poses additional challenges, particularly because anisotropic conduction, three-dimensional tissue architecture, and physiologically occurring conduction pathways need to be considered. To address this point, we revised the Discussion and no longer explicitly refer to organoids as the primary next model system (see lines 548-560). Instead, we now discuss 3D cardiac constructs more generally and specifically mention tissue-engineered heart muscle and cardiac slice cultures as intermediate models for future validation. In particular, cardiac slice cultures are advantageous in this context because they preserve the native three-dimensional cellular and fiber architecture and are therefore expected to better maintain anisotropic conduction behavior than current organoid models. We further clarify in the revised Discussion that clinical translation will require homogeneous expression of an optogenetic tool and additional adaptation of the sparse-sampling-based wavefront characterization approach to account for anisotropic conduction in 3D tissue.

12. Please make sure to label/explain all variables used in the Figures in the Figure legends. In Figure 2fh please label the x-axes of the graphs provided

We thank the reviewer for pointing this out. We have revised the figure legends to ensure that all variables used in the figures are defined and explained. In addition, we have labeled the x-axes in Figure 2f,h as requested.

13. Please correct spelling mistakes/typos

We have carefully proofread the revised manuscript and corrected any spelling mistakes or typographical errors identified during revision.

The authors thank the reviewers for their valuable comments.

On behalf of all authors,
Robert Wendland

Response to the Reviewers

Nature Communications Engineering submission,
Manuscript ID: COMMSENG-26-0039,
Type: research article

Title: All-Optical Closed-Loop Control of Human Cardiomyocyte Networks Exploiting Holographic Optogenetics

Authors: Robert Wendland, Felix Schmieder, Muhammad Atif Sikandar, Frithjof Paul Knüppel, Wolfram-Hubertus Zimmermann, Olaf Bergmann, Lars Büttner, Jürgen Czarske

black: Comments of the Reviewers
blue: the authors´ reply to these comments

Please note that the referenced text passages refer to the revised manuscript (**with markup**).

Reviewer 1:

The authors have made a substantial effort to address the reviewers' concerns. In particular, the added simulation-based analysis of ROI number, ROI geometry, noise sensitivity, wavefront curvature, and achievable cycle time strengthens the methodological basis of the sparse-sensing framework. However, one concern remains insufficiently resolved. The added analyses do not fully address the need for an appropriate open-loop baseline. The rebuttal explains why open-loop stimulation is expected to be less robust, but this is not equivalent to a controlled comparison using the same directional-correction endpoint. The manuscript should either include a matched open-loop comparison, even if limited, or substantially soften claims that the closed-loop strategy provides superior spatiotemporal regulation. In summary, the revision improves the manuscript, I would recommend a minor revision before acceptance.

We agree that the added open-loop pacing analysis does not constitute a matched open-loop comparison using the same directional-correction endpoint. To address this concern, we have revised the corresponding Discussion paragraph and substantially softened the claims regarding the benefit of closed-loop control. Specifically, we no longer state that the closed-loop strategy provides superior spatiotemporal regulation compared with an open-loop approach. Instead, we now describe the rationale for using feedback-guided stimulation in the present proof-of-concept implementation (see lines 504-511).

Reviewer 2:

Thank you for the thorough revision. Most concerns have been satisfactorily addressed.

Two minor textual additions I think would help the final version: (1) an explicit acknowledgement that the spatial uniformity of the electromechanical delay was not measured, and that spatially heterogeneous delay could bias propagation angle estimates; and (2) inclusion of the quantitative phase delay error at tachycardia rates (~52% at 250 bpm) discussed in the rebuttal but absent from the manuscript.

I do not anticipate a further round of review.

We thank the reviewer for the positive assessment of the revised manuscript and for pointing out these two remaining textual clarifications. We have now incorporated both points into the Discussion. First, we now explicitly acknowledge that the magnitude and spatial uniformity of the electromechanical delay were not quantitatively assessed in the present study. We further clarify that spatial heterogeneities in this delay could bias mechanical-motion-derived estimates of electrical propagation, including the propagation angle (see lines 430 and 436). Second, we have added the quantitative phase-delay error at tachycardia-relevant rates to the Discussion. Specifically, we now state that the relative phase-delay error decreases with increasing sampling frequency but increases at higher contraction frequencies and may therefore become substantial at tachycardia-relevant rates. To make this limitation quantitative, we included the previously discussed illustrative estimate of approximately 52% at 250 bpm for the current 60 Hz camera-based implementation (see lines 568-575).

Reviewer 3:

The authors have carefully revised the manuscript, addressing all points raised during the initial review.

We thank the reviewer for the positive assessment of our revised manuscript and are pleased that the revisions have addressed the points raised during the initial review.

The authors thank the reviewers for their valuable comments.

On behalf of all authors,
Robert Wendland
