# Supplementary material for: All-optical closed-loop control of human cardiomyocyte networks exploiting holographic optogenetics
Source: Commun Eng. 2026 Sep 17;5:159. doi: 10.1038/s44172-026-00779-1 (PMC13586264; doi:10.1038/s44172-026-00779-1)
Supplement: Supplementary file 2 [file 44172_2026_779_MOESM2_ESM.pdf]

Supplementary Information

"All-Optical Closed-Loop Control of Human Cardiomyocyte Networks Exploiting Holographic Optogenetics"

Robert Wendland,[a,b,*] Felix Schmieder,[a,b] Muhammad Atif Sikandar,[c,d] Frithjof Paul Knüppel,[c] Wolfram-Hubertus Zimmermann,[c,e,f,g,h,i] Olaf Bergmann,[c,e,j] Lars Büttner,[a,b] Jürgen W Czarske[a,b,k,l,m]

a Laboratory of Measurement and Sensor System Techniques, TUD Dresden University of Technology, Helmholtzstraße 18, 01062 Dresden, Germany

b Competence Center BIOLAS, TUD Dresden University of Technology, Helmholtzstraße 18, 01062 Dresden, Germany

c Department of Pharmacology and Toxicology, University Medical Center Göttingen, Robert-Koch-Str. 40, 37075 Göttingen, Germany

d TUD Dresden University of Technology, 01062 Dresden, Germany

e Deutsches Zentrum für Herz-Kreislauf-Forschung (DHKZ) partner site Lower Saxony, Göttingen, Germany

f Cluster of Excellence "Multiscale Bioimaging: from Molecular Machines to Networks of Excitable Cells" (MBExC), University of Göttingen, Germany

g German Center for Neurodegenerative Diseases (DZNE), Göttingen, Germany

h German Center for Child and Adolescent Health (DZKJ), Göttingen, Germany

i Fraunhofer Institute for Translational Medicine and Pharmacology (ITMP), Göttingen, Germany

j Karolinska Institutet, CMB, 171 77 Stockholm, Sweden

k Wyant College of Optical Sciences, University of Arizona, Tucson, Arizona, United States

l Institute of Applied Physics, Faculty of Science, TUD Dresden University of Technology, Dresden, Germany

m Excellence Cluster Physics of Life, TUD Dresden University of Technology, Dresden, Germany

Correspondence: (*) Robert.Wendland@tu-dresden.de

Table of contents

Supplementary note 1 Optical Setup for Label-Free Imaging and Holographic Stimulation

Supplementary note 2 Characterization of Optical Stimulation Efficiency

Supplementary note 3 Monitoring of the Contraction Activity and Beating Frequency Measurement

Supplementary note 4 Contraction Wavefront Visualization and Activity Map Generation

Supplementary note 5 Real-Time Contraction Rhythm Extraction from Sparsely Sampled Mechanical Activity

Supplementary note 6 Quantitative Real-Time Analysis of Planar Wavefronts Based on Sparsely Sampled Mechanical Activity

Supplementary note 7 Simulation of the Optical Setup for Label-Free Imaging

Supplementary note 8 Phase Delay Measurement Error

Supplementary note 9 Validation of Phase Delay Measurements

Supplementary note 10 Maximum Distance of a Detectable Rotor Core Based on Phase Delay Measurements

References

Supplementary note 1: Optical Setup for Label-Free Imaging and Holographic Stimulation

The complete optical setup is visualized schematically in supplementary figure S1a. The stimulation path is highlighted in yellow, while the imaging path is visualized in red. To realize holographic stimulation of the f-Chrimson[1] modified human cardiomyocytes, a fiber-coupled (Thorlabs P5-405BPM-FC-2; Thorlabs PAF2A-18A) laser (MPB Communications 2RU-VFL-Series, λ=589 nm; 200 mW) is used. The collimated beam is expanded by the lenses L1 (Thorlabs AC254-030-A) and L2 (Thorlabs AC508-150-A) to illuminate a fast binary ferroelectric SLM (Forth Dimension Displays QXGA-3DM M175). The half wave plate λ/2 (Thorlabs WPH10M-588) is used to ensure the SLM is illuminated in its preferred polarization angle. Pinhole PH1 (Thorlabs VRC4CPT) acts as spatial frequency filter located in the Fourier plane between lenses L1 and L2. The SLM displays binary phase only Fourier computer-generated holograms (CGHs), generated as described in the Methods section of this paper. The holograms' first diffraction order is selected as the stimulation source of the sample using a rectangular aperture A (homebuilt, 0-3 cm × 0-4 cm). The binary SLM allows polarization shifts of approx. 60°. The linear polarizer LP (Thorlabs LPVISE200-A) is used to project the polarization of the light coming from the SLM onto its polarization axes, yielding a binary 180° phase modulation. The lens L3 (Thorlabs ACT508-250-A) performs the transformation of the holograms from the Fourier domain. The resulting stimulation pattern is magnified and projected onto the sample S by the lenses L4 (lens combination of Thorlabs AC508-080-A and Thorlabs LC1611-A) and L5 (Thorlabs AC508-150-A). To acquire widefield images of the cardiomyocytes, a 660 nm LED (Thorlabs M660L4) together with lens L6 (Thorlabs AC254-030-A) is integrated into the setup. The beam is aligned with the stimulation beam on the optical axis by a dichroitic mirror DM (Thorlabs DMSP605R). To realize the tracking of contraction wavefronts label-free, a 660 nm laser source (Quantum MPC6000 IGNIS 660) is coupled into a fiber (Thorlabs S1FCA, Thorlabs FT030-Y), whose output is obliquely mounted above the sample (see Fig. S1b). In front of the fiber output a diffusor D (Thorlabs DG10-600) is mounted to realize homogenous illumination of the sample in the FOV. The oblique laser illumination results in speckle patterns, changing with the contractions of the sample. These speckle patterns are projected onto the camera C (IDS uEye UI-3040SE-M-GL) by the lenses L7 (Thorlabs AC508-150-A) and L8 (Thorlabs AC508-075-A). The pinhole PH2 (Thorlabs ID25) allows for spatial filtering in the Fourier plane. To stop stimulation light from reaching the camera, a laser line filter LF (2x Thorlabs NF594-23) is mounted in front of the

camera. All mirrors are Thorlabs PF10-03-F01 or Thorlabs PF20-03-F01. The PC used for executing closed loop control and data analysis uses an Intel Core i5-9500 CPU at 3.00 GHz as well as 16 GB of RAM at 2667 MHz.

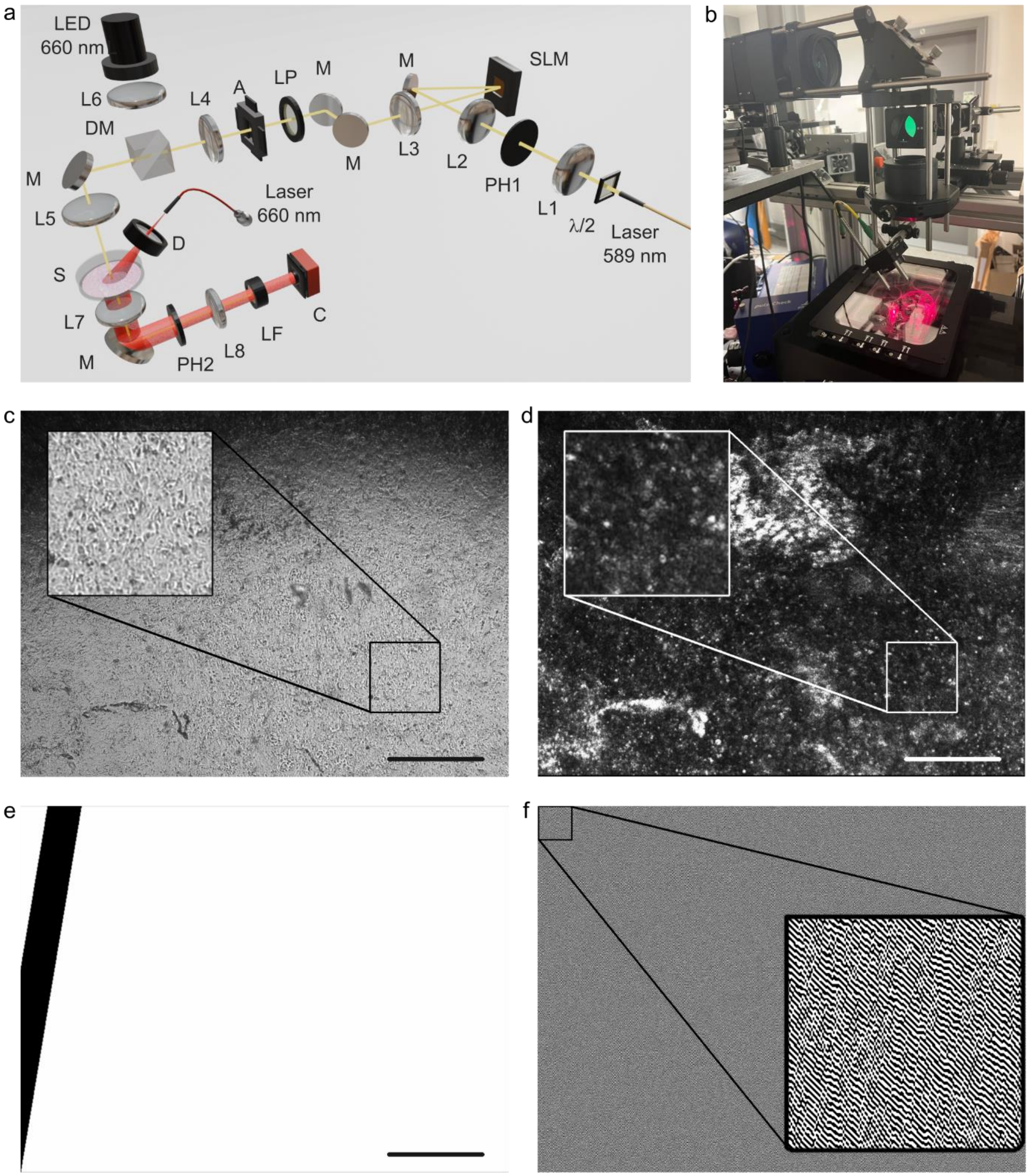

Supplementary Figure S1 Setup and exemplary images for data acquisition and conduction of experiment. (a) Schematic of the complete setup. The schematic was created using assets from the Optical Components Pack v1 by Ryo Mizuta Graphics. (b) Representative image of the samples in the incubator during an

experiment. (c) Widefield image of LED-illuminated cardiomyocyte monolayer. (d) Speckle pattern resulting from oblique laser illumination at the same center wavelength. (e) desired stimulation pattern, the angled black bar denotes the illuminated area. (f) Computer-generated binary phase hologram to create the pattern shown in (d). All insets magnified from the small framed areas. Scale bars: 2 mm.

The all-optical tracking of contraction wavefronts relies on contraction-induced local changes of the intensity of speckles created by 660 nm laser light passing the investigated cell networks, an approach similar to temporal difference speckle methods[2]. As shown in supplementary figure S1c, widefield illumination with 660 nm provides little contrast for contractions, especially for increasing FOVs, since intensity changes get mapped to ever less pixels. Supplementary figure S1d shows an example of the speckle contrast achieved with our setup for the same location on the same sample as in supplementary figure S1c, resulting in better dynamic range of intensity changes on the camera. Experiment-specific stimulation patterns are generated as images denoting the desired locations for stimulation marked in black as in supplementary figure S1e. For the calculation of the wavefront-shaping holograms, these images are converted to complex matrices by adding a random phase to each pixel. Then, images are transformed according to Eq. 3, taking into account the physical properties of our setup, and then Fourier-transformed and binarized. This process results in a computer-generated hologram as in panel f in supplementary figure S1, where the inset shows a magnified portion of the hologram. Scale bars for all marked images represent a distance of 2 mm.

Supplementary note 2: Characterization of Optical Stimulation Efficiency

We first characterized the stimulation efficiency of cardiomyocytes by exposing a round culture patch of $A_{\mathrm{stim}} = 0.56\ \mathrm{mm}^2$ for $t_{\mathrm{stim}} = 100\ \mathrm{ms}$ to increasing levels of intensity I_{stim} from 0 to 5 mW/mm^2, measured at the sample plane, which resulted in guaranteed contractions starting from 3.75 mW/mm^2. Similarly, we tested the dependency of the contraction on the duration of the stimulus t_{stim} by exposing 0.56 mm^2 culture patches to increasing durations of 3.75 mW/mm^2 light stimuli ranging from 10 ms to 200 ms, resulting in guaranteed contractions starting from 150 ms. Both experiments can be summarized using the radiant exposure $H_{\mathrm{stim}} = I_{\mathrm{stim}} \cdot t_{\mathrm{stim}} / A_{\mathrm{stim}}$ required to elicit contractions, as can be seen in supplementary figure S2a. Both data agree well with an inverse exponential fit, suggesting that in the investigated regime the energy supplied to the cells is the crucial parameter. According to the fit, a 95 % probability to stimulate contraction can be achieved by illuminating cell cultures with a radiant exposure of approx. 380 µJ/mm^2.

Further, we determined rheobase and chronaxie of our cell cultures[3,4]. Rheobase is defined as the "minimal [impulse strength] of infinite duration leading to excitation". Chronaxie is the excitation pulse duration resulting in an excitation for a pulse strength of twice the rheobase (both from Ref. 3). We determined these parameters by increasing the stimulation intensity for each stimulation time (stimulation frequency $\mathrm{f}_{\mathrm{stim}} = 1\ \mathrm{Hz}$) until we elicited a response in N=6 samples. Here, an illumination area of 3.14 mm^2 was used. The results of these experiments can be seen in supplementary figure S2b as mean ± standard deviation indicated in blue. The minimum and maximum stimulation intensity for each stimulation time is indicated by the gray-shaded area. Fitting a Weiss-Lapicque-curve $I_{\mathrm{stim}} = I_{\mathrm{rheo}}(1 + \tau / t_{\mathrm{stim}})$ to the data[3,4], we can extract the rheobase $I_{\mathrm{rheo}} = 54.5\ \mu\mathrm{W/mm}^2$ and chronaxie $\tau = 123.3\ \mathrm{ms}$.

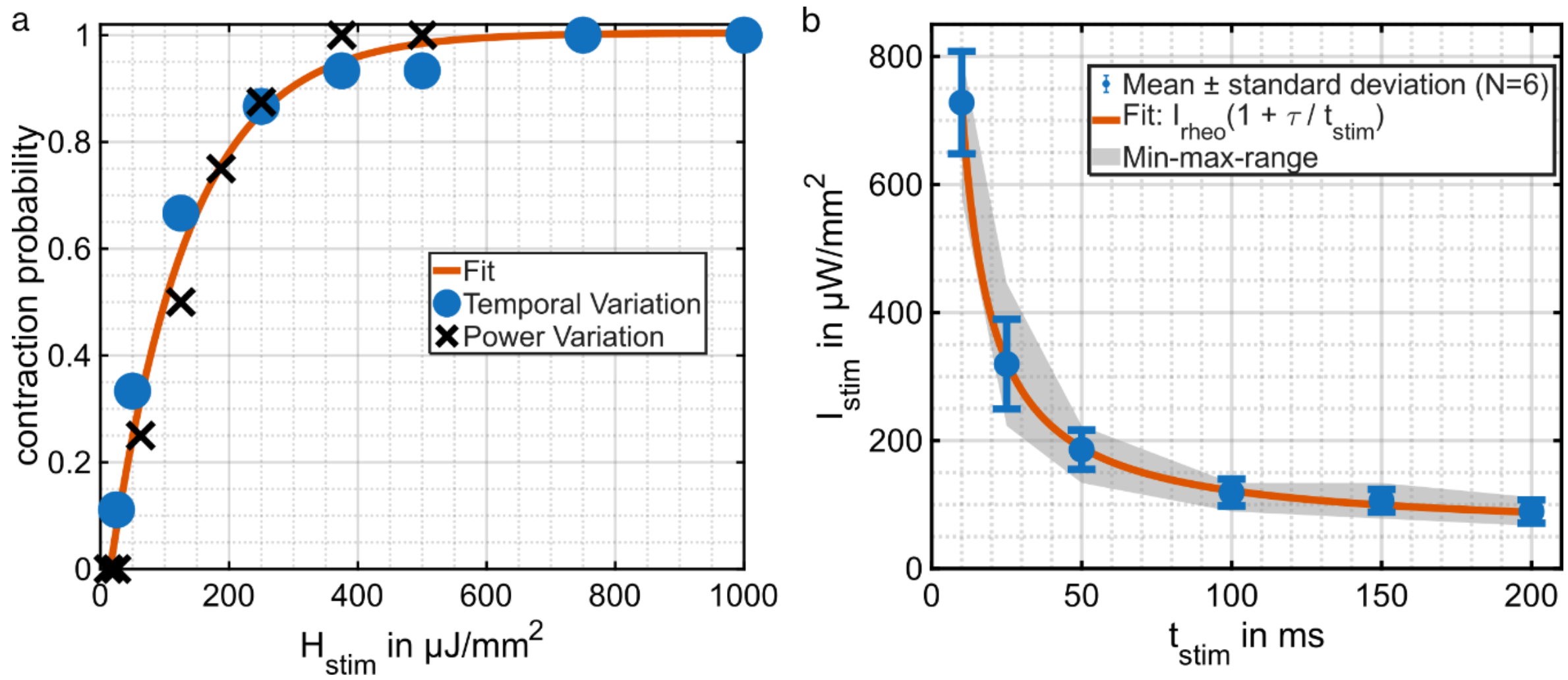

Supplementary Figure S2 Characterization of contraction response as a function of optical stimulation strength. (a) Probability of eliciting cell contraction as a function of radiant exposure used for stimulation, summarizing two independent experiments altering the stimulation power and duration, respectively, for a constant size of the illuminated area of 0.56 mm^2. Dots show constant power data with the time being varied; crosses show constant time data with power being varied. Red line shows an inverse exponential fit to which all data agree well (mean squared error MSE $= 2.5 \cdot 10^{-3}$). (b) Stimulation intensity I_{stim} over stimulation time t_{stim} for the determination of rheobase I_{rheo} and chronaxie τ of Chrimson-expressing human cardiomyocyte monolayers. Blue dots and error bars indicate mean and standard deviation measured over N=6 samples. Minimum and maximum stimulation intensities required for each stimulation time are indicated by gray shading. Orange curve represents the fit of the Weiss-Lapicque-curve $I_{\mathrm{stim}} = I_{\mathrm{rheo}}(1 + \tau/t_{\mathrm{stim}})$. All experiments were conducted using an illumination area of 3.14 mm^2.

We further investigated the effect of varying stimulation frequencies on the ability to elicit cell contractions sequences (see supplementary figure S3). Here, stimulation frequencies f_{stim} were selected as multiples of the spontaneously occurring contraction frequency f_{nat} of the respective cell culture. Across N=141 experiments, we observed spontaneous contraction frequencies ranging from 0.16 Hz to 2 Hz with a median of 0.85 Hz. Supplementary figure 3 shows the elicited contraction frequency f_{con} over the stimulation frequency, both normalized to the spontaneous frequency. All cultures following the linear function $f_{\mathrm{con}}/f_{\mathrm{nat}} = a \cdot f_{\mathrm{stim}}/f_{\mathrm{nat}} + b$ with $a = 1$ and $b = 0$ show a 1:1 coupling of reaction to stimulation, whereas data on the function with $a = 0$ and $b = 1$ do not react to stimulation at all. As can be seen, stimulation below the spontaneously occurring contraction frequency is largely ineffective. However, for very low

spontaneous frequencies, some cultures react to every second ($a = 1/2$) or third ($a = 1/3$) stimulus, see supplementary figure S3a.

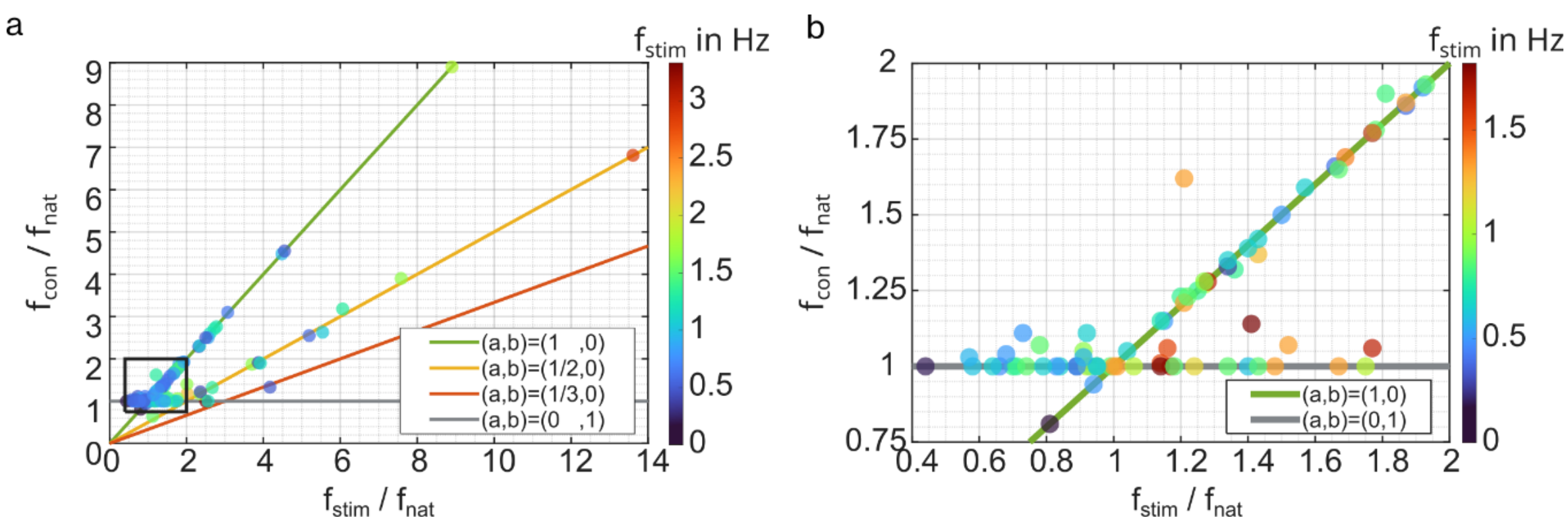

Supplementary Figure S3 Characterization of contraction response as a function of stimulation frequency. (a) Observed contraction frequency f_{con} over stimulation frequency f_{stim}, both normalized to the spontaneously occurring contraction frequency f_{nat}. Color codes the stimulation frequency in Hz. (b) Magnified section of (a), visualized by the black box in (a). Several separate regimes can be observed, divided into four regimes following linear functions $f_{\mathrm{con}}/f_{\mathrm{nat}} = a \cdot f_{\mathrm{stim}}/f_{\mathrm{nat}} + b$: Cultures following every stimulation ($a = 1; b = 0$), every second ($a = 1/2\,; b = 0$) or every third ($a = 1/3\,; b = 0$) stimulus and cultures not following stimulations at all ($a = 0; b = 1$). For all experiments an $H_{\mathrm{stim}} = 195\ \mu\mathrm{J/mm^2}$ was used. Note that this low radiant exposure level may be the explanation why not all datapoints lay on the straight lines with positive slopes for $f_{\mathrm{stim}}/f_{\mathrm{nat}} > 1$.

Chrimson and its variants have, to the day of the writing of this manuscript, a more widespread application as a tool for the manipulation of neural populations with a recent increase in use in cardiology, in some instances combined with other tools for simultaneous stimulation and inhibition[1,5-12]. Stimulus response observation methods vary between direct observation of action potentials[1,6,9,10] and indirect macroscopic responses like muscle contractions[7,8], which, in addition to the varying Chrimson variants with different kinetics, complicates a direct comparison with the results of this study. Compared to directly stimulated neurons (0.75 μJ/mm^2 to 100 μJ/mm^2), f-Chrimson-activated cardiomyocytes in our study require higher energies to elicit 1:1 pacing. In comparison to indirectly activated muscle tissue, our samples required either comparable radiant exposure, around 300 μJ/mm^2 (Ref. 8), or less, as values up to 500 mJ/mm^2 have been reported for indirect activation[7]. Studies also investigating the direct optical stimulation of f-Chrimson found much lower requirements with 0.1 μJ/mm^2 using atrial-engineered human myocardium[11]. Other studies using the opsin Channelrhodopsin-2 (ChR2) found that stimulating larger areas of samples

results in comparably lower required stimulation intensities[13], a fact which is reflected by our results shown in supplementary figure S2. This suggests that in future experiments, larger areas could be illuminated to lower response thresholds, if necessary, i.e. to limit possible effects of phototoxicity. Regarding the chronaxie of our monolayers, the values we find are approximately two to three times as high as reported for Chrimson as expressed in the construct BiPoles in human cardiomyocyte monolayers[14]. Compared to Ref. 11, the rheobase value we find is half as high (54 µW/mm^2 vs. 118 µW/mm^2) at much higher chronaxie (123 ms vs. 5 ms), which is likely due to differences in the number of stimulated cells[15].

Since our setup for the optical detection of contraction waves uses 660 nm light, there is an overlap with the action spectrum of Chrimson at roughly 20 % of the maximum (extrapolated from Ref. 1 and Ref. 9) which might lead to subthreshold excitation of our samples. As a consequence, stimulation thresholds reported here might be too low and refractory periods of the cells are possibly higher than without illumination[16], possibly leading to lower achievable maximum stimulation frequencies.

Supplementary note 3: Monitoring of the Contraction Activity and Beating Frequency Measurement

The contraction activity of the cell cultures was measured by extracting a one-dimensional time signal from videos of contraction-induced temporal speckle fluctuations. The procedure is summarized in supplementary algorithm A1. It relies on evaluating the difference between each video frame F and a slowly varying background B, which is computed as the temporal mean within a window of size $(2 \cdot L_{\mathrm{BG}} + 1)$ to account for general intensity changes. We set $2 \cdot L_{\mathrm{BG}} + 1 = 101$, corresponding to 1683 ms in our configuration. The absolute difference to the background AD is summed up over all pixels to obtain values of the time trace I_{raw}. A Tukey apodization window TW (75 % taper width) is applied to I_{raw}, yielding the windowed signal I_{win}. The signal is then transformed into the frequency domain, denoted by a hat ($\hat{I}_{\mathrm{win}}$), where a second-order Butterworth bandpass filter BP is applied to remove low- and high- frequency artefacts unrelated to cardiomyocyte contractions. The filter was parametrized with a lower cutoff frequency of 0.2 Hz and an upper cutoff frequency of 3 Hz.

Supplementary algorithm A1: Contraction activity extraction from speckle patterns

Input: video recording of speckle pattern F

Output: normalized time signal I corresponding to the contraction activity of the monolayers

FOR each video frame $F(m, n, i)$

  update background: $B(n_x, n_y, i) = \frac{1}{2 \cdot L_{\mathrm{BG}} + 1} \sum_{i-L_{\mathrm{BG}}}^{i+L_{\mathrm{BG}}} F(n_x, n_y, i)$

  calculate absolute difference: $AD(n_x, n_y, i) = \left| F(n_x, n_y, i) - B(n_x, n_y, i) \right|$

  sum up all pixels: $I_{\mathrm{raw}}(i) = \sum_{\forall (n_x, n_y)} AD(n_x, n_y, i)$

ENDFOR

apply Tukey window: $I_{\mathrm{win}}(i) = I_{\mathrm{raw}}(i) \cdot TW(i)$

transform into frequency domain: $\hat{I}_{\mathrm{win}}(f) = \mathcal{F}\{I_{\mathrm{win}}(i)\}$

apply bandpass filter: $\hat{I}_{\mathrm{filtered}}(f) = \hat{I}_{\mathrm{win}}(f) \cdot BP(f)$

inverse transformation into time domain:	$I_{\text{filtered}}(i) = \mathcal{F}^{-1}\{\hat{I}_{\text{filtered}}(f)\}$
normalize signal:	$I(i) = \underset{\forall i}{\text{norm}}[I_{\text{filtered}}(i)]$

Algorithm 1: Contraction activity extraction from speckle patterns. i denotes a discrete time stamp, f a frequency component, and n_x and n_y denote the horizontal and vertical pixel indices within a frame.

This procedure enables the extraction of the contraction activity as can be seen exemplarily in Fig. 1d. Since the shape of the contraction peaks may vary, we calculated the beating frequency not from the maxima but based on the temporal distance between the maxima of the temporal derivative of the time signal I, which is plotted in Fig. 1e.

Supplementary note 4: Contraction Wavefront Visualization and Activity Map Generation

The image processing procedure for visualizing contraction wavefronts using our label-free imaging approach is summarized in supplementary algorithm A2. The goal is to derive a map of isochrones showing the progress of a contraction wave in the FOV during each contraction-relaxation-cycle. Each pixel of the map represents the time of the first passing of the wavefront at this location. To generate these maps, first, video frames F are divided into square boxes of 20 pixel in width. From each box, a time signal I_{box} is extracted following supplementary algorithm A1. Aggregating pixels into larger boxes increases the likelihood of capturing representative local intensity changes while reducing the influence of unresponsive or noisy pixels. The temporal derivative of each box's time signal, denoted D_{box}, is approximated using a fourth-order central finite difference scheme. Negative values are suppressed through thresholding to isolate contraction events ($D_{\mathrm{box}} > 0$), yielding D^{+}_{box}. The result is a set of frames of derivatives F_{der} of local intensity changes. Next, the frames are smoothed in the spatial domain using a Gaussian kernel G with a full width at half maximum (FWHM) of 35 pixels. Finally, the smoothed frames F_{smo} are normalized to the range $[0\,,1]$ over the last $L_{\mathrm{norm}} = 140$ values, resulting in F_{res}, which visualizes the cardiac contraction wavefronts.

Supplementary algorithm A2: Contraction wavefront visualization based on contraction induced intensity changes

Input: video recording of speckle pattern F

Output: video with visible contraction wavefronts F_{res}

```
subdivide video frames F(n_x, n_y, i) into boxes
FOR each box
    apply algorithm 1                       I_box(i)
    approximate temporal derivative:        D_box(i) ~ I_box(i-2) - 8·I_box(i-1)
                                                       +8·I_box(i+1) - I_box(i+2)
```

$$D_{\mathrm{box}}(i) \sim I_{\mathrm{box}}(i-2) - 8 \cdot I_{\mathrm{box}}(i-1) + 8 \cdot I_{\mathrm{box}}(i+1) - I_{\mathrm{box}}(i+2)$$

eliminate negative values:	$D_{\text{box}}^{+}(i) = \max_{\forall i}[D_{\text{box}}(i), 0]$
assign to spatial position	$F_{\text{der}}(n_x, n_y, i)$
**ENDFOR**	
**FOR** each derivative frame $F_{\text{der}}(m, n, i)$	
spatial smoothing:	$F_{\text{smo}}(n_x, n_y, i) = G(n_x, n_y) * F_{\text{der}}(n_x, n_y, i)$
**ENDFOR**	
normalization:	$F_{\text{res}}(n_x, n_y, i) = \underset{i-L_{\text{norm}} \leq i' \leq i}{\text{norm}}[F_{\text{smo}}(n_x, n_y, i')]$

Supplementary algorithm A2: Contraction wavefront visualization based on contraction induced intensity changes. i denotes a discrete time stamp, and n_x and n_y denote the horizontal and vertical pixel indices within a frame.

To reduce the computational effort of the following steps, only frame stacks from the processed video F_{res} containing contraction events were analyzed. The mechanical activity was determined using supplementary algorithm A1, and the start and end points of each contraction were identified from the extrema of its temporal derivatives. For each contraction stack, an activity map was generated as outlined in supplementary algorithm A4. First, the frames were binarized (supplementary figure S4a). If the total excited area in one frame did not exceed a predefined threshold (described below), the content of this frame was merged with the next one. Connected pixel regions ("objects") were then identified, their areas calculated, and sorted in descending order (supplementary figure S4b). To limit computation, only the 20 largest objects were retained. From these, only upper outliers exceeding a minimum area threshold defined as $A_{\text{obj}} \geq A_{\text{mean}} + \frac{1}{3}[A_{\text{max}} - A_{\text{mean}}]$ were considered as part of the wavefront, where A_{mean} is the mean area and A_{max} the maximum area within this subset (supplementary figure S4c,d). When the total excited area exceeded a threshold, morphological dilation (disk-shaped structuring element, radius = 4 px) was applied to connect wavefront segments (supplementary figure S4e). Holes within the wavefront were filled (supplementary figure S4f), and the inverse image was also filled to remove

residual artefacts not connected to the wavefront (supplementary figure S4g). The result was smoothed using a Gaussian kernel (FWHM = 40 px). If regions of the FOV had not previously

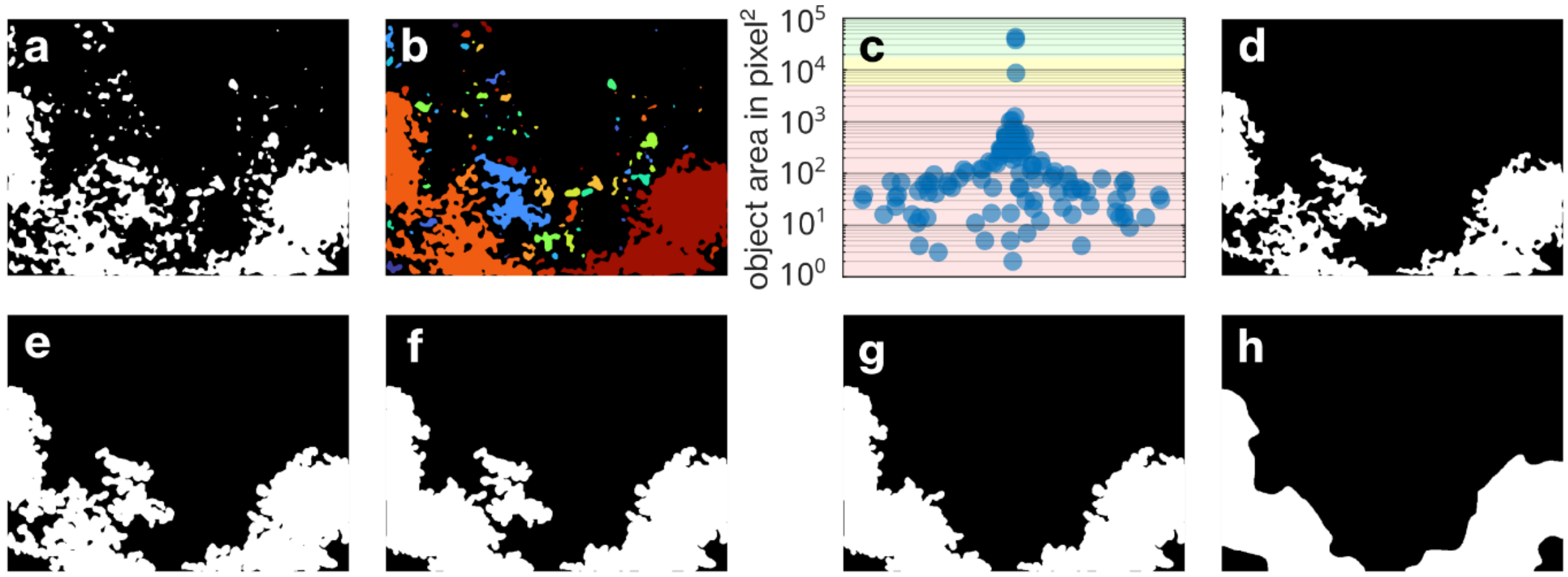

Supplementary Figure S4 Image processing steps for wavefront shape extraction. (a) Processed frame F_{res} after binarization. (b) Identified objects in the binarized frame labeled in different colors. (c) Distribution of the areas of the objects from (b); retained large-area objects are highlighted in green; for demonstration purposes, additional retained objects are highlighted in yellow; discarded objects are highlighted in red. (d) Remaining objects in frame after area-thresholding. (e) Frame after dilation. (f) Frame after filling operation. (g) Frame after removal of the filled inverse. (h) Smoothed wavefront shape.

been active, the corresponding time stamp was written to the respective location in the isochrone map (supplementary figure S4h). This process was repeated for each frame in the contraction stack, yielding a temporary map (supplementary figure S5a).

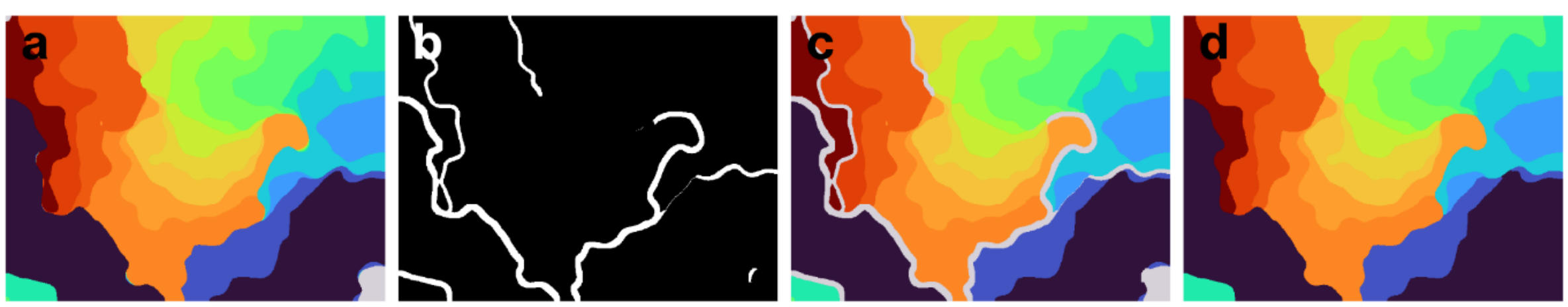

Supplementary Figure S5 Image processing steps for isochrone map generation. (a) Superposed isochrone map extracted from a frame stack corresponding to a single contraction peak. (b) Regions with high local standard deviation are marked in white. (c) Isochrone map after removal of the regions identified in (b). (d) Final isochrone map.

In this map, pixels with a local standard deviation greater than $5\ /\ f_{\mathrm{cam}}$ were removed, where f_{cam} denotes the camera frame rate (supplementary figure S5b,c). The local standard deviation was calculated within an 11×11 px window. This threshold reflects the assumption that wavefronts propagate continuously, with each sampling step ($1\ /\ f_{\mathrm{cam}}$) yielding a follow-up image

of the wavefront located alongside the previous one. Remaining artefacts smaller than a minimum area threshold were removed. Pixels with missing values were finally filled by extending the borders of neighboring wavefronts into the corresponding regions (supplementary figure S5d), yielding the final activity map of the contraction event.

Supplementary algorithm A3: Creation of activity maps

Input: videos with visualized wavefronts F_{res} and time signal with mechanical activity signal I

Output: activity maps depicting contraction timing within the FOV for each contraction

calculate temporal derivatives of I
find extrema in derivatives
derive frame stacks within a contraction event
FOR each frame stack
    FOR each frame within stack
        binarize video frames F_{res}
        (optional: cumulate with last frame)
        identify connected pixels (objects)
        FOR each object
            calculate area
        ENDFOR
        sort objects by area
        consider 20 largest objects
        search for upper outliers and remove other objects
        IF total excited area $<$ minimal excited area
            cumulate with next frame
        ELSE

```
            dilate objects
            filling operation
            remove artefacts, not connected to the wavefront
            spatial smoothing
            insert wavefront in temporary map with corresponding time stamp
        ENDIF
    ENDFOR
    localize pixels with high local standard deviation in temporary map and replace
    them with NaNs
    FOR each wavefront in temporary map
        calculate area of the wavefront
        IF area < minimal structure size
            remove wavefront and replace with NaNs
        ENDIF
    ENDFOR
    interpolate areas of NaNs
    save final map of contraction activity
ENDFOR
```

Supplementary algorithm A3: Creation of activity maps. Note that in case of a rotating contraction frame stacks are derived from a local mechanical activity.

Supplementary note 5: Real-Time Contraction Rhythm Extraction from Sparsely Sampled Mechanical Activity

To reduce computation time and enable real-time operation, time signals are extracted only from a limited set of predefined regions of interest (ROIs) within the field of view. Recorded videos are hardware-binned by a factor of two to further decrease processing load. The general principle of signal extraction follows the fundamental procedure described in Algorithm 1 and is summarized here in supplementary algorithm A4. For each ROI p of the currently recorded camera frame (i), the corresponding image segment F_p is extracted from the speckle pattern. This segment is then used to update the background estimate B_p for every ROI. Unlike in the offline approach, only preceding frames are available in the real-time setting. Therefore, a cumulative mean is employed for background estimation. Once B_p is estimated, the current value of the time signal $I_{p,\mathrm{raw}}$ is computed as the pixelwise sum of absolute differences with respect to the background. Due to the sensitivity of the laser-speckle-based label-free imaging approach to externally induced motion—particularly external vibrations—the extracted signals are smoothed, yielding $I_{p,\mathrm{smo}}$. This is achieved through an online convolution using a boxcar kernel BC of length L_{conv}, which introduces a temporal delay between contraction activity and its detection of $(L_{\mathrm{conv}} - 1)/2$ frames (assuming L_{conv} is odd). A kernel length of 19 frames was selected, resulting in a delay of 150 ms. Following smoothing, the resulting signal I_p is obtained by normalizing $I_{p,\mathrm{smo}}$ to the range $[0\,,1]$ relative to the preceding $L_{\mathrm{norm}} = 180$ frames. The binary activity state of each ROI is then determined by thresholding the normalized signal at $I_{\mathrm{th}} = 1/2$. The timestamps of positive threshold crossings t_p are extracted and further used to analyze the propagation of the contraction wavefront.

Supplementary algorithm A4: Real-time capable signal extraction from ROIs

Input: current frame F, captured by the camera, activity threshold I_{th}

Output: time signals I_p corresponding to the mechanical activity and contraction times t_p for each ROI

1 **FOR** each ROI p

extract ROI image segment from F	$F_p(n_x, n_y, i)$
update background:	$B_p(n_x, n_y, i) = \frac{1}{i}\left[\frac{B_p(n_x, n_y, i-1)}{i-1} + F_p(n_x, n_y, i)\right]$
extract time signal:	$I_{p,\mathrm{raw}}(i) = \sum_{\forall(n_x, n_y)} \lvert F_p(n_x, n_y, i) - B_p(n_x, n_y, i) \rvert$
smooth signal:	$I_{p,\mathrm{smo}}(i) = I_{p,\mathrm{raw}}(i) * BC(L_{\mathrm{conv}})$
normalize signal:	$I_p(i) = \underset{i-L_{\mathrm{norm}} \leq i' \leq i}{\mathrm{norm}}\left[I_{p,\mathrm{smo}}(i')\right]$
**ENDFOR**	
threshold signals with I_{th}	t_p

Supplementary algorithm A4: Real-time capable signal extraction from ROIs. i denotes a discrete time stamp, and n_x and n_y denote the horizontal and vertical pixel indices within a frame.

Supplementary note 6: Quantitative Real-Time Analysis of Planar Wavefronts Based on Sparsely Sampled Mechanical Activity

Supplementary algorithm 4 enables the acquisition of time stamps of contraction at distinct spatial locations. By modeling the contraction propagation as a plane wave, this enables the determination of characteristics, like propagation angle and wavefront velocity. If the wavefront passes through two ROIs p and q, located at $\vec{r}_p$ and $\vec{r}_q$, at times t_p and t_q with the velocity v_c the temporal lag $\Delta t_{p,q} = [t_p - t_q]$ fulfills:

$$\vec{k} \cdot [\vec{r}_p - \vec{r}_q] = v_c \cdot |\vec{k}| \cdot \Delta t_{p,q} \,, \tag{S1}$$

where $\vec{k}$ denotes the wave vector. Using multiple ROIs, a linear system of equations,

$$\underline{A} \cdot \vec{b} = \vec{c} \quad , \text{ with:} \tag{S2a}$$

$$\underline{A} = \begin{bmatrix} \vec{r}_1 - \vec{r}_2 \\ \vdots \\ \vec{r}_p - \vec{r}_q \\ \vdots \end{bmatrix}, \qquad \vec{b} = \frac{\vec{k}}{|\vec{k}|} \cdot \frac{1}{v_c} \text{ and } \vec{c} = \begin{bmatrix} \Delta t_{1,2} \\ \vdots \\ \Delta t_{p,q} \\ \vdots \end{bmatrix}, \tag{S2b}$$

is created, where $\vec{b}$ is estimated by a minimum-norm least squares solution, yielding the propagation direction $\tan(\vartheta) = b_y / b_x$. The velocity of the wavefront v_c is then obtained from:

$$v_c = \frac{s_{p,q}}{\Delta t_{p,q}} \quad , \text{ with: } s_{p,q} = (\vec{r}_p - \vec{r}_q) \cdot \begin{pmatrix} \cos(\vartheta) \\ \sin(\vartheta) \end{pmatrix}, \tag{S3}$$

where $s_{p,q}$ is the projection of the inter-ROI distance onto the propagation direction.

Supplementary note 7: Simulation of the Optical Setup for Label-Free Imaging

To systematically validate and assess limitations of the ROI-based sparse sensing approach (described in detail in section ”*Wavefront Characteristics from Sparsely Sampled Mechanical Activity*”, supplementary note 5 and 6), we simulated the optical setup described in supplementary note 1. The general idea is to simulate motion-induced intensity changes in the recorded speckle patterns based on predefined regions representing areas of cellular contraction. Subsequently, time traces—forming the basis of the ROI-based measurement approach—will be extracted, and the measured parameters will be compared with the simulation input. The simulation environment for motion-induced changes in speckle patterns is illustrated schematically in Fig. S6.

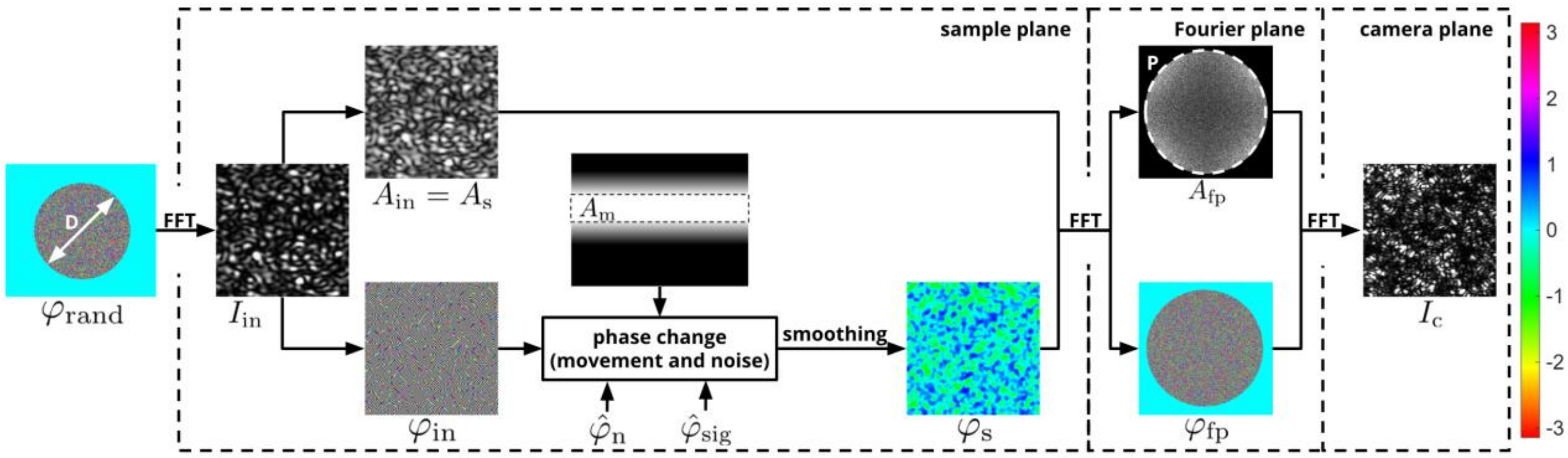

Supplementary Figure S6 Random phase values, φ_{rand}, are assigned within a circular region of diameter D. A Fourier transform is then used to generate a static speckle pattern in the sample plane, described by the input amplitude A_{in} and phase φ_{in}. To simulate motion, the phase φ_{in} is locally modulated within a defined modulation area A_{m}, with a maximum motion-induced phase change of $\hat{\varphi}_{\mathrm{sig}}$. Noise is introduced by adding random phase variations across the full field, with a maximum noise-induced phase change of $\hat{\varphi}_{\mathrm{n}}$. The resulting complex field in the sample plane, $A_{\mathrm{s}} \cdot \exp(\mathrm{j} \cdot \varphi_{\mathrm{s}})$, including both motion-induced and noise-related phase variations, is propagated to the Fourier plane. In the Fourier plane, a circular pupil function P is applied, yielding the complex field $A_{\mathrm{fp}} \cdot \exp\left(\mathrm{j} \cdot \varphi_{\mathrm{fp}}\right)$. This field is subsequently propagated to the camera plane, where the simulated camera intensity distribution I_{c} is obtained.

First, a static speckle pattern is created with the approach described in Ref. 17: A circle with a diameter $D = 500$ px is filled with uniformly distributed random phase values φ_{rand}. The speckle pattern, corresponding to the intensity in the sample plane I_{in} is then calculated as:

$$I_{\mathrm{in}} = \underline{E}_{\mathrm{in}} \cdot \underline{E}_{\mathrm{in}}^{*} \quad \text{with:} \quad \underline{E}_{\mathrm{in}} = \mathcal{F}\left\{e^{\mathrm{j} \cdot \varphi_{\mathrm{rand}}}\right\}. \qquad \text{(S4)}$$

Here, $\underline{E}_{\mathrm{in}} = A_{\mathrm{in}} \cdot e^{\mathrm{j} \cdot \varphi_{\mathrm{in}}}$ denotes the initial simulated complex electrical field in the sample plane. The whole simulated plane has the dimensions $(759\ \mathrm{px}) \times (1012\ \mathrm{px})$, where the FOV covers $(543\ \mathrm{px}) \times (724\ \mathrm{px})$. To simulate motion within a defined area, a mask A_{m} defining the area of

sample motion is used. The binary mask is smoothed using a gaussian kernel. The phase values of φ_{in} where the smoothed mask has values greater than zero are varied and the difference to the initial phase value is scaled with the respective value of the smoothed mask. Further, all values of φ_{in} are additionally varied to model different noise levels (see Eq. S6). The electrical field in the sample plane $\underline{E}_s = A_{\mathrm{s}} \cdot e^{\mathrm{j} \cdot \varphi_{\mathrm{s}}}$ consists of:

$$A_{\mathrm{s}} = A_{\mathrm{in}} \ , \tag{S5}$$

$$\varphi_{\mathrm{s}} = \varphi_{\mathrm{in}} + \left[\hat{\varphi}_{\mathrm{sig}} \cdot 2\pi \cdot U(0,1)\right]_{|\mathrm{motion}} + \hat{\varphi}_{\mathrm{n}} \cdot 2\pi \cdot U(0,1)\ . \tag{S6}$$

The symbol $U(0,1)$ denotes uniformly distributed random values between 0 and 1, and $\hat{\varphi}_{\mathrm{sig}}$ and $\hat{\varphi}_{\mathrm{n}}$ indicate the maximum phase changes due to the movement (signal) and noise respectively. To eliminate numerical artefacts of later Fourier transforms, the phase is smoothed using a Gaussian kernel with a standard deviation of 1 px. The field $\underline{E}_s$ is then propagated to the Fourier plane of the 4f-system, where the point spread function of the optical system is taken into account as multiplication with a circular pupil function P. Finally, the intensity distribution on the camera I_{c} is calculated via the Fourier transform as:

$$I_{\mathrm{c}} = \underline{E}_{\mathrm{c}} \cdot \underline{E}_c^* \quad \text{with:} \quad \underline{E}_c = \mathcal{F}\left\{P \cdot \mathcal{F}\{\underline{E}_{\mathrm{s}}\}\right\}. \tag{S7}$$

To validate the simulation environment, spontaneous contractions in a cardiomyocyte monolayer are evaluated using both label-free and ROI-based sparse sensing approaches, with the aim of sufficiently reproducing the experimental results through simulation of the optical setup. In Fig. S7a,b is the speckle pattern recorded during the experiment (left) compared with the simulated speckle pattern (right). The main observable differences are the intensity gradient and certain structural features in the experimental image that are not included in the simulation. However, as these are sample-specific and as long as the ROIs are sufficiently illuminated, they do not affect algorithm performance, since time traces corresponding to the motion of the cardiomyocytes can still be extracted. The activation map, shown in Fig. S7c corresponds to a spontaneous contraction of the cardiomyocyte monolayer.

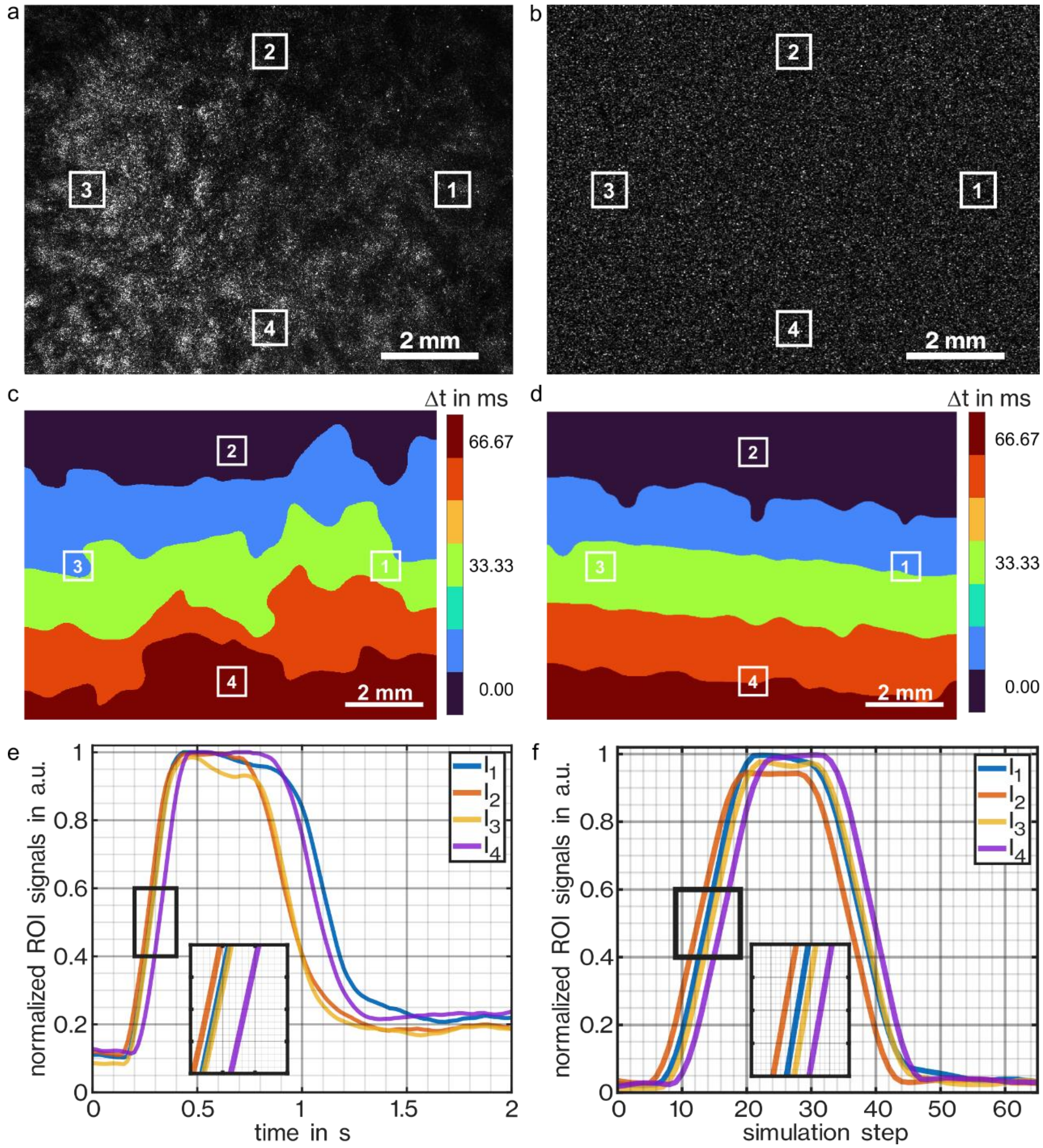

Supplementary Figure S7 Comparison of an experimentally recorded spontaneous contraction with a simulated plane wave propagation. (a) Speckle pattern, acquired with the optical system. (b) Simulated speckle pattern. (c) Activity map derived from experimental measurement. (d) Activity map obtained from a simulated contraction using experimentally measured parameters. White boxes indicate the positions of ROIs (e) ROI signals I_1 to I_4 recorded during the experiment. (f) ROI signals obtained from the simulated setup.

Wavefront parameters were measured based on time traces extracted from the ROIs indicated as white boxes in the activity map. The propagation angle ϑ_{exp} was measured as 265° and the wavefront velocity $v_{\mathrm{c,exp}}$ as 91 mm/s from the temporal delay of the rising edges of the four ROI

signals, shown in Fig. S7e. To mimic the dynamics of the plane wave, A_{m} was set to a stripe moving through the simulated FOV with speed $v_{\mathrm{c,exp}}$ and orientation ϑ_{exp}. Then, the simulated speckle movement was processed using the same algorithm as for the experimental data. The resulting activity map is shown in Fig. S7d. As expected, the movement steps of the visualized wavefront are similar to the experiment, visualized in Fig. S7c. Only slight differences, originating from the assumption of an ideal plane wavefront can be seen, when comparing Fig. S7c with Fig. S7d. Also, time signals from the same ROIs were extracted from the simulation (Fig. S7f). Based on these time traces, the propagation angle and wavefront velocity were measured, yielding an orientation of the wavefront $\vartheta_{\mathrm{sim}} = 263°$ and a velocity of $v_{\mathrm{c,sim}}$ of $90\ \mathrm{mm/s}$, showing only a little difference compared to the experiment. When comparing the simulated time traces with Fig. S7e, the experimental traces show slight differences in peak shape. In particular, time traces I_1 and I_3 exhibit a small decrease during the plateau phase whereas the simulated traces remain close to their maximum. The shape of these experimental time traces is related to the prolonged contraction of the cardiomyocytes. During this time, the monolayers remain tense and the difference relative to the ROI-specific moving background approximation may decrease. This can lead to the local minima or slight decreases of the time traces in Fig. S7e after the first maxima. This does not pose a problem for evaluating the performance of the ROI-based measurement approach with the simulation, since the measurement only relies on the rising edges of the recorded time signals which corresponds to the propagation of the contraction wavefronts (not relaxation). As can be seen from the comparison of the rising edges of the time traces, the inputs for the measurement, namely the activation order of the ROIs and the respective temporal differences, are well matched between simulation and experiment. Therefore, the simulation environment proves to be suited to investigate limitations of the proposed ROI-based techniques, as well as for general investigations that are described in section *"Wavefront Characteristics from Sparsely Sampled Mechanical Activity"*.

Further, we estimated the practically relevant amplitude ratios $\hat{\varphi}_{\mathrm{n}}/\hat{\varphi}_{\mathrm{sig}}$. For this purpose, we analyzed no-contraction time windows in the experimentally recorded contraction-derived ROI signals, assuming that the remaining signal fluctuations during these intervals predominantly represent noise. We then compared the corresponding standard deviations with those obtained from simulations performed with defined noise amplitude ratios, as described above. For the

simulated signals, we found an approximately linear relationship between the imposed noise-to-signal amplitude ratio and the resulting standard deviation σ_I of the time traces ($R^2 = 0.97$):

$$\hat{\varphi}_{\mathrm{n}}/\hat{\varphi}_{\mathrm{sig}}\,(\sigma_I) = 8.681 \cdot \sigma_I - 0.016\,. \qquad \text{(S7)}$$

Using this regression model, we estimated the experimental noise-to-signal amplitude ratios from the standard deviations σ_I measured during no-contraction time windows in the experiments presented in Fig. 2. One of the 13 experiments was excluded from this analysis because the available no-contraction interval was very short, which would have resulted in a high uncertainty of the estimated σ_I. Therefore, this analysis was performed with $N = 12$ experiments. The resulting experimental noise-to-signal amplitude ratio was $\hat{\varphi}_{\mathrm{n}}/\hat{\varphi}_{\mathrm{sig}} = 0.23 \pm 0.11$ (mean ± standard deviation).

Supplementary note 8: Phase Delay Measurement Error

The maximum relative error in the phase delay measurement between two points p and q, resulting from uncertainties in the detection of the respective activation times, can be derived from Eq. 1 using standard error propagation:

$$\left|\frac{\Delta\left(\hat{\varphi}_{p,q}\right)}{\Delta\varphi_{p,q}}\right| = \frac{1}{\left|\Delta\varphi_{p,q}\right|}\left[\left|\frac{\partial\left(\Delta\varphi_{p,q}\right)}{\partial t_p}\cdot\Delta\hat{t}_p\right| + \left|\frac{\partial\left(\Delta\varphi_{p,q}\right)}{\partial t_q}\cdot\Delta\hat{t}_q\right| + \left|\frac{\partial\left(\Delta\varphi_{p,q}\right)}{\partial T}\cdot\Delta\hat{T}\right|\right], \qquad \text{(S9)}$$

where hats (^) denote maximum possible errors. The maximum uncertainty in activation time detection is the same for both points ($\left|\Delta\hat{t}_p\right| = \left|\Delta\hat{t}_q\right| = |\hat{t}|$). The period T is calculated as the time difference between two consecutive contractions at a single location and therefore doubly impacted by the error in activation time detection ($\left|\hat{T}\right| = 2\cdot|\hat{t}|$), which is determined by the frame rate of the camera ($|\hat{t}| = 1/f_{\text{cam}}$). Applying this and expressing $\left|\Delta t_{p,q}\right|$ in terms of the projected distance between p and q in the direction of wave propagation and the wavefront velocity v_c, supplementary equation (S4) leads to the final formulation of the maximum relative error:

$$\left|\frac{\Delta\left(\Delta\hat{\varphi}_{p,q}\right)}{\Delta\varphi_{p,q}}\right| = \frac{2}{f_{\text{cam}}}\cdot\left|\frac{v_c}{R_{p,q}\cdot\cos\left(\vartheta-\alpha_{p,q}\right)} + \frac{1}{T}\right|, \qquad \text{(S10)}$$

where $R_{p,q}$ is the Euclidian distance between p and q, ϑ is the direction of wave propagation and $\alpha_{p,q}$ the angle of the vector connecting p and q. In supplementary equation (S5), the error of phase delay measurement is influenced, on the one hand, by the parameters v_c and T, which arise from the biophysical properties of the cardiomyocytes. On the other hand, controllable factors such as the chosen alignment of the ROIs and, above all, the sampling rate also affect the error. The sampling rate is of particular importance, as the measurement error is inversely proportional to it. Therefore, f_{cam} offers a powerful means to reduce uncertainty. Assuming our hardware and ROI alignment as shown in Fig. 2b, we set $R_{1,2} = 4.7\text{ mm}$, $\alpha_{1,2} = 127°$ and $f_{\text{cam}} = 60\text{ Hz}$. For illustration we further use exemplarily values of v_c and T and $\left|\Delta\varphi_{1,2}\right|$ obtained from one experiment in Fig. 2g,h, yielding $v_c = 70\text{ mm/s}$, $T = 3.57\text{ s}$ and $\left|\Delta\varphi_{1,2}\right| = 6.7\,°$. The corresponding propagation angle was $\vartheta = -39\,°$. Inserting all this in supplementary equation (S4), we get $\left|\Delta\left(\Delta\hat{\varphi}_{1,2}\right)\right| = 3.36°$. Although the relative error may appear large in comparison to $\left|\Delta\varphi_{1,2}\right| = 6.7\,°$, it is important to note that the primary objective is to distinguish planar and rotating waves, where phase delays typically exceed single-digit values. Another noteworthy aspect is that the maximum relative error approaches infinity when the planar wave propagates

perpendicular to the interconnection of ROIs p and q. In this case, a phase delay may not be detected if $\left|\Delta t_{p,q}\right| < 1/f_{\mathrm{cam}}$. However, this does not pose a practical limitation for wavefront classification: the phase delay $\left|\Delta\varphi_{p,q}\right|$ is then effectively treated as zero, leading to the correct identification of a planar wavefront within our framework.

Supplementary note 9: Validation of Phase Delay Measurements

The phase delay $\Delta\varphi_{p,q}$ between two points p and q can be extracted from the temporal delay $\Delta t_{p,q}$ of a wavefront, passing these points ($\Delta\varphi_{p,q} = 360° \cdot \Delta t_{p,q}/T$) relative to the contraction period T. This phase delay depends on several parameters. In the following, we describe the influence of both biological properties of cardiomyocytes and factors related to the ROI alignment. One influential parameter is the contraction period T, the inverse of the contraction frequency f_{con}. Since $\Delta\varphi_{p,q}$ is calculated relative to T, a longer contraction period results in a smaller phase delay, and *vice versa*. In cardiac monolayers, the contraction period is strongly affected by environmental factors such as temperature. Importantly, although T may vary, it is bounded from below by the effective refractory period (ERP) of the cardiomyocytes. Beyond the contraction frequency, the propagation velocity v_c of the contraction wavefronts also influences the measured phase delay. Variations in v_c may arise from sample inhomogeneities. Assuming that T and the ROI alignment remain constant, an increase in v_c reduces the measured phase delay between two points. The phase delay further depends on the interplay between biological properties and ROI alignment. Specifically, the relative angle between the straight line connecting ROIs p and q and the propagation angle $\vartheta - \alpha_{p,q}$ has impact on $\Delta\varphi_{p,q}$. In addition, the spatial separation $R_{p,q}$, which is directly determined by the ROI alignment, also affects the phase delay: greater spacing yields larger delays if all other parameters remain constant. These dependencies are summarized in the following table.

**Parameter**	**Impact on phase delay**
period T	longer contraction period leads to a decrease of the phase delay, since: $$\Delta\varphi_{p,q} \sim \frac{1}{T}\,.$$
propagation velocity v_c	higher propagation velocity leads to a decrease of the phase delay, since: $$\Delta\varphi_{p,q} \sim \Delta t_{p,q} \sim 1/v_c\big|_{s_{p,q}=\mathrm{const.}}\,.$$
angle difference $\vartheta - \alpha_{p,q}$	change of the propagation direction affects the effective (projected) distance $s_{p,q}$ between the observation points and therefore: $$\Delta\varphi_{p,q} \sim \Delta t_{p,q} \sim s_{p,q}\big|_{v_c=\mathrm{const.}} \sim \cos(\vartheta - \alpha_{p,q})\,.$$
spatial distance $R_{p,q}$	increased distance between the observation points leads to an increase of the phase delay, since:

	$\Delta\varphi_{p,q} \sim \Delta t_{p,q} \sim s_{p,q}\big\vert_{v_c=\text{const.}} \sim R_{p,q}.$

Although many parameters vary and influence the measured phase delay, a phase delay threshold for distinguishing planar and rotational waves remains reasonable, since the variation of these parameters is limited, especially of the period T by the ERP. However, to validate the phase delay measurements between sparsely sampled signals across different experiments, a scaling of the measured phase delay with respect to the varying parameters is necessary. This scaled phase $\Delta\varphi^*_{p,q}$ can be derived as:

$$\Delta\varphi^*_{p,q} = \frac{T \cdot v_c}{R_{p,q}} \cdot \Delta\varphi_{p,q} = 360° \cdot \cos\left(\vartheta - \alpha_{p,q}\right), \tag{S11}$$

and is corrected with respect to the contraction wave parameters (T, v_c) as well as the geometrical arrangement of the ROI $(R_{p,q})$, enabling the validation of measured contraction wavefront characteristics. The angle between the ROI p and q (Fig. 2a) is denoted by $\alpha_{p,q}$. We then plotted the scaled phase measurements against the measured propagation angle and compared it with the theoretical expectation (right side of Eq. S11). The result is shown in supplementary figure S8. Each group of four vertically aligned data points belongs to one experiment, where propagation angle, velocity, period, and phase delay for each ROI with respect to ROI 1 were measured. The close agreement between the data and the theoretical model (mean average error MAE = 18.16°, equivalent to 5 % of the scaled phase amplitude) confirms the validity of our measurement approach and the resulting data.

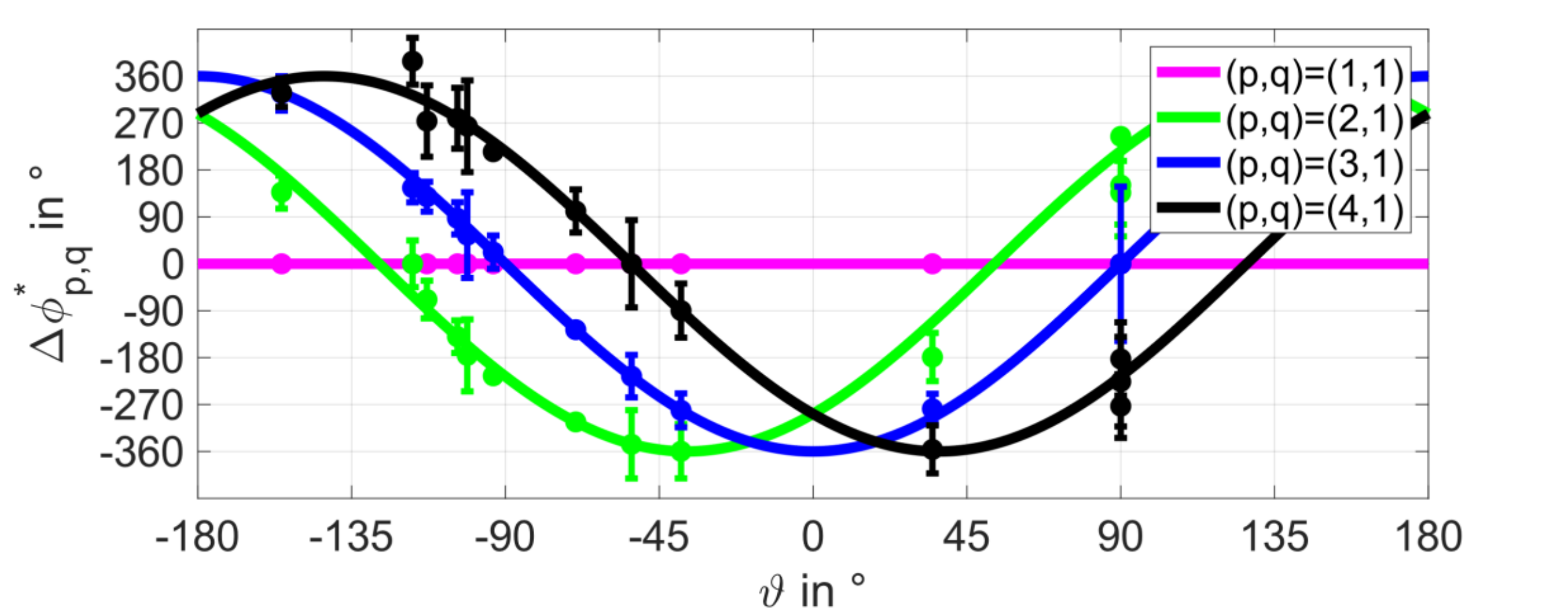

Supplementary Figure S8 Scaled phase (see Eq. 11) for ROI pairs (p, q) plotted as median ± standard deviation over the measured propagation angle, based on multiple contractions of one cell culture; error bars

denote standard deviation. Solid lines indicate theoretical model of scaled phase: $\Delta\varphi^{*}_{p,q} = 360° \cdot \cos(\vartheta - \alpha_{p,q})$.

Supplementary note 10: Maximum Distance of a Detectable Rotor Core Based on Phase Delay Measurements

To quantify the detection limit of a rotational wave, we used a simple model, an uncurved wavefront rotating around $(x_\mathrm{c}|y_\mathrm{c})$. Due to the symmetry of the ROI placement (diamond shape, as described in the main section), it is sufficient to observe only one quadrant of the coordinate system. The maximum phase delay,

$$\Delta\varphi_{p,q}^{\max} = \max_{p,q\,\in\,\mathcal{M}_\mathrm{ROI}} \left|\Delta\varphi_{p,q}\right| \text{, with: } \mathcal{M}_\mathrm{ROI} = \{1, 2, 3, 4\}\,, \tag{S12}$$

measured between two ROIs p and q as a function of the rotor core position $(x_\mathrm{c}|y_\mathrm{c})$, normalized by the maximum distance d_max between any ROI and the central reference point $(x_\mathrm{O}|y_\mathrm{O})$ is visualized in Fig. 2i.

References

1. Mager, T. *et al.* High frequency neural spiking and auditory signaling by ultrafast red-shifted optogenetics. *Nat. Commun.* **9**, 1750 (2018). https://doi.org/10.1038/s41467-018-04146-3

2. Martí-López, L., Cabrera, H., Martínez-Celorio, R. A. & González-Peña, R. Temporal difference method for processing dynamic speckle patterns. *Opt. Commun.* **283**, 4972–4977 (2010). https://doi.org/10.1016/j.optcom.2010.07.073

3. Gorbunov, B. B. *et al.* A history of the discovery of the Hoorweg–Weiss–Lapicque law. *Biomed. Eng.* **52**, 357–360 (2019). https://doi.org/10.1007/s10527-019-09847-7

4. Irnich, W. The chronaxie time and its practical importance. *Pacing Clin. Electrophysiol.* **3**, 292–301 (1980). https://doi.org/10.1111/j.1540-8159.1980.tb05236.x

5. Mukherjee, R. K. *et al.* Successful identification of and discrimination between atrial and ventricular arrhythmia with the aid of pacing and defibrillator devices. *Arrhythm. Electrophysiol. Rev.* **10**, 235–240 (2021). https://doi.org/10.15420/aer.2021.54

6. Vierock, J. *et al.* BiPOLES is an optogenetic tool developed for bidirectional dual-color control of neurons. *Nat. Commun.* **12**, 4527 (2021). https://doi.org/10.1038/s41467-021-24759-5

7. Schild, L. C. & Glauser, D. A. Dual color neural activation and behavior control with Chrimson and CoChR in *Caenorhabditis elegans. Genetics* **200**, 1029–1034 (2015). https://doi.org/10.1534/genetics.115.177956

8. Murawski, C. *et al.* Segment-specific optogenetic stimulation in *Drosophila melanogaster* with linear arrays of organic light-emitting diodes. *Nat. Commun.* **11**, 6248 (2020). https://doi.org/10.1038/s41467-020-20013-6

9. Klapoetke, N. C. *et al.* Independent optical excitation of distinct neural populations. *Nat. Methods* **11**, 338–346 (2014). https://doi.org/10.1038/nmeth.2836

10. Li, L. *et al.* Colocalized, bidirectional optogenetic modulations in freely behaving mice with a wireless dual-color optoelectronic probe. *Nat. Commun.* **13**, 839 (2022). https://doi.org/10.1038/s41467-022-28539-7

11. Seibertz, F. *et al.* Atrial fibrillation-associated electrical remodelling in human induced pluripotent stem cell-derived atrial cardiomyocytes: a novel pathway for antiarrhythmic therapy development. *Cardiovasc. Res.* **119**, 2623–2637 (2023). https://doi.org/10.1093/cvr/cvad143

12. Schneider, L. V. *et al.* Bioengineering of a human innervated cardiac muscle model. *Preprint at bioRxiv* (2023). https://doi.org/10.1101/2023.08.18.552653

13. Junge, S., Schmieder, F., Sasse, P., Czarske, J., Torres-Mapa, M. L. & Heisterkamp, A. Holographic optogenetic stimulation with calcium imaging as an all optical tool for cardiac electrophysiology. *J. Biophotonics* e202100352 (2022). https://doi.org/10.1002/jbio.202100352

14. Schwarzová, B. *et al.* Modulating cardiac physiology in engineered heart tissue with the bidirectional optogenetic tool BiPOLES. *Pflugers Arch.* **475**, 1463–1477 (2023). https://doi.org/10.1007/s00424-023-02869-x

15. Bruegmann, T. *et al.* Optogenetic control of heart muscle *in vitro* and i*n vivo*. Nat. Methods **7**, 897–900 (2010). https://doi.org/10.1038/nmeth.1512

16. Biasci, V. *et al.* Optogenetic manipulation of cardiac electrical dynamics using sub-threshold illumination: dissecting the role of cardiac alternans in terminating rapid rhythms. *Basic Res. Cardiol.* **117,** 25 (2022). https://doi.org/10.1007/s00395-022-00933-8

17. Duncan, D. D. &, Kirkpatrick, S.J. Algorithms for simulation of speckle (laser and otherwise), *Proc. SPIE* 6855, Complex Dynamics and Fluctuations in Biomedical Photonics V, 685505 (2008). https://doi.org/10.1117/12.760518
