# Supplementary material for: All-optical closed-loop control of human cardiomyocyte networks exploiting holographic optogenetics
Source: Commun Eng. 2026 Sep 17;5:159. doi: 10.1038/s44172-026-00779-1 (PMC13586264; doi:10.1038/s44172-026-00779-1)
Supplement: Supplementary file 3 [file 44172_2026_779_MOESM3_ESM.pdf]

nature portfolio

Corresponding author(s): DAPR, COMMSENG-26-0039

Last updated by author(s): Feb 6, 2026

Reporting Summary

Nature Portfolio wishes to improve the reproducibility of the work that we publish. This form provides structure for consistency and transparency in reporting. For further information on Nature Portfolio policies, see our Editorial Policies and the Editorial Policy Checklist.

Statistics

For all statistical analyses, confirm that the following items are present in the figure legend, table legend, main text, or Methods section.

n/a	Confirmed	
☐	☒	The exact sample size (*n*) for each experimental group/condition, given as a discrete number and unit of measurement
☐	☒	A statement on whether measurements were taken from distinct samples or whether the same sample was measured repeatedly
☐	☒	The statistical test(s) used AND whether they are one- or two-sided<br>*Only common tests should be described solely by name; describe more complex techniques in the Methods section.*
☒	☐	A description of all covariates tested
☐	☒	A description of any assumptions or corrections, such as tests of normality and adjustment for multiple comparisons
☐	☒	A full description of the statistical parameters including central tendency (e.g. means) or other basic estimates (e.g. regression coefficient) AND variation (e.g. standard deviation) or associated estimates of uncertainty (e.g. confidence intervals)
☐	☒	For null hypothesis testing, the test statistic (e.g. *F*, *t*, *r*) with confidence intervals, effect sizes, degrees of freedom and *P* value noted<br>*Give P values as exact values whenever suitable.*
☒	☐	For Bayesian analysis, information on the choice of priors and Markov chain Monte Carlo settings
☐	☒	For hierarchical and complex designs, identification of the appropriate level for tests and full reporting of outcomes
☐	☒	Estimates of effect sizes (e.g. Cohen's *d*, Pearson's *r*), indicating how they were calculated

Our web collection on statistics for biologists contains articles on many of the points above.

Software and code

Policy information about availability of computer code

Data collection	Matlab version 2023b, Matlab version 2025a, Microsoft Excel version 2016, uEye Cockpit version 4.96.0650
Data analysis	Matlab and toolboxes: Curve Fitting Toolbox, Image Acquisition Toolbox, Image Processing Toolbox<br>The self-developed Matlab codes that support findings of this study are available from the corresponding author (by mail) upon reasonable request, although restrictions may apply due to privacy reasons and ongoing research projects.

For manuscripts utilizing custom algorithms or software that are central to the research but not yet described in published literature, software must be made available to editors and reviewers. We strongly encourage code deposition in a community repository (e.g. GitHub). See the Nature Portfolio guidelines for submitting code & software for further information.

Data

Policy information about availability of data

All manuscripts must include a data availability statement. This statement should provide the following information, where applicable:

- Accession codes, unique identifiers, or web links for publicly available datasets
- A description of any restrictions on data availability
- For clinical datasets or third party data, please ensure that the statement adheres to our policy

The data and material that support the findings of this study are available upon reasonable request to the corresponding authors.

Research involving human participants, their data, or biological material

Policy information about studies with human participants or human data. See also policy information about sex, gender (identity/presentation), and sexual orientation and race, ethnicity and racism.

Reporting on sex and gender	n/a
Reporting on race, ethnicity, or other socially relevant groupings	n/a
Population characteristics	n/a
Recruitment	n/a
Ethics oversight	n/a

Note that full information on the approval of the study protocol must also be provided in the manuscript.

Field-specific reporting

Please select the one below that is the best fit for your research. If you are not sure, read the appropriate sections before making your selection.

☒ Life sciences ☐ Behavioural & social sciences ☐ Ecological, evolutionary & environmental sciences

For a reference copy of the document with all sections, see nature.com/documents/nr-reporting-summary-flat.pdf

Life sciences study design

All studies must disclose on these points even when the disclosure is negative.

Sample size	In general, the sample size corresponds to number of different cell cultures, utilized for the experiments.<br>If this is not the case, this is explicitly indicated in the text.
Data exclusions	Experiments with low signal-to-noise ratios arising from experimental limitations (e.g., imaging conditions or externally induced motion) or from a lack of detectable cellular response to optical stimulation, rather than reflecting biological effects, were excluded .
Replication	All reported results were derived from experiments performed on at least six independent cell cultures.
Randomization	Randomization for different experimental groups was not applicable as experiments were performed on uniform biological material, i.e. genetically modified cardiac cell cultures.
Blinding	Blinding to group allocation was not applicable, as no group allocation was performed.

Reporting for specific materials, systems and methods

We require information from authors about some types of materials, experimental systems and methods used in many studies. Here, indicate whether each material, system or method listed is relevant to your study. If you are not sure if a list item applies to your research, read the appropriate section before selecting a response.

Materials & experimental systems

n/a	Involved in the study	
☒	☐	Antibodies
☐	☒	Eukaryotic cell lines
☒	☐	Palaeontology and archaeology
☒	☐	Animals and other organisms
☒	☐	Clinical data
☒	☐	Dual use research of concern
☒	☐	Plants

Methods

n/a	Involved in the study	
☒	☐	ChIP-seq
☒	☐	Flow cytometry
☒	☐	MRI-based neuroimaging

Eukaryotic cell lines

Policy information about cell lines and Sex and Gender in Research

Cell line source(s)	TC-1133 human pluripotent cells, Human CD34+ umbilical cord blood cell derived induced pluripotent stem cells, Lonza (comercially available)
Authentication	We did not authenticate the TC-1133 cell line
Mycoplasma contamination	all cells have been tested for Mycoplasma contamination
Commonly misidentified lines (See ICLAC register)	none

Plants

Seed stocks	n/a
Novel plant genotypes	n/a
Authentication	n/a
